# A Rab escort protein regulates the MAPK pathway that controls filamentous growth in yeast

**DOI:** 10.1038/s41598-020-78470-4

**Published:** 2020-12-17

**Authors:** Sheida Jamalzadeh, Atindra N. Pujari, Paul J. Cullen

**Affiliations:** 1 Department of Chemical and Biological Engineering, University at Buffalo, State University of New York, Buffalo, NY USA; 2 Department of Biological Sciences, State University of New York at Buffalo, 532 Cooke Hall, Buffalo, NY 14260-1300 USA

**Keywords:** Functional genomics, Fungal genomics

## Abstract

MAPK pathways regulate different responses yet can share common components. Although core regulators of MAPK pathways are well known, new pathway regulators continue to be identified. Overexpression screens can uncover new roles for genes in biological processes and are well suited to identify essential genes that cannot be evaluated by gene deletion analysis. In this study, a genome-wide screen was performed to identify genes that, when overexpressed, induce a reporter (*FUS1-HIS3*) that responds to ERK-type pathways (Mating and filamentous growth or fMAPK) but not p38-type pathways (HOG) in yeast. Approximately 4500 plasmids overexpressing individual yeast genes were introduced into strains containing the reporter by high-throughput transformation. Candidate genes were identified by measuring growth as a readout of reporter activity. Fourteen genes were identified and validated by re-testing: two were metabolic controls (*HIS3*, *ATR1*), five had established roles in regulating ERK-type pathways (*STE4*, *STE7*, *BMH1*, *BMH2*, *MIG2*) and seven represent potentially new regulators of MAPK signaling (*RRN6*, *CIN5*, *MRS6*, *KAR2, TFA1, RSC3, RGT2*). *MRS6* encodes a Rab escort protein and effector of the TOR pathway that plays a role in nutrient signaling. *MRS6* overexpression stimulated invasive growth and phosphorylation of the ERK-type fMAPK, Kss1. Overexpression of *MRS6* reduced the osmotolerance of cells and phosphorylation of the p38/HOG MAPK, Hog1. Mrs6 interacted with the PAK kinase Ste20 and MAPKK Ste7 by two-hybrid analysis. Based on these results, Mrs6 may selectively propagate an ERK-dependent signal. Identifying new regulators of MAPK pathways may provide new insights into signal integration among core cellular processes and the execution of pathway-specific responses.

## Introduction

During cell differentiation, cells specialize into specific types by the action of signal transduction pathways. Mitogen-activated protein kinase (MAPK) pathways control numerous responses, including cell differentiation, proliferation, cell migration, and apoptosis^1,2^. MAPK pathways control diverse responses by regulating the expression of a large number of target genes. There are four types of MAPK pathways: RAF-MEK-ERK1/2, JNK1/2/3, p38α/β/γ/δ, and ERK5^1,3^. Remarkably, these pathways can share common components, which leads to proper cross-talk in normal settings and unregulated cross-talk in the disease state. Mis-regulation of MAPK signaling leads to inappropriate responses, such as cancers and problems with immune system function^4–6^. Due to the crucial roles of MAPK pathways in regulating fundamental cellular processes, they remain the focus of investigation by many labs and are a focus for therapeutic targeting^7–12^.

MAPK pathways are evolutionarily conserved signaling modules in eukaryotes, and fundamental insights into MAPK pathway regulation have come from studies in many systems. The budding yeast *Saccharomyces cerevisiae* is a unicellular organism that has been extensively used as a model for studying signaling pathways^7,13–19^. Like in other eukaryotes, yeast utilizes ERK-type and p38-type MAPK pathways^20,21^. One ERK-type pathway mediates the response to nutrient-limiting conditions that permit filamentous (pseudohyphal/invasive) growth, a fungal-type foraging response resulting in the formation of chains of elongated interconnected cells^22,23^. This pathway functions through a set of kinases that function in a tandem series: p21 activated [PAK] Ste20 (MAPKKKK), Ste11 (MAPKKK), Ste7 (MAPKK), and Kss1 (MAPK)^24,25^. A second ERK-type pathway in yeast controls the mating of haploid cells through an almost identical set of kinases: Ste20 (PAK), Ste11 (MAPKKK), Ste7 (MAPKK), and Fus3 and Kss1 (MAPK). Two MAPKs, Fus3, and Kss1, function in mating and filamentous growth pathways, respectively. It has been shown that the deletion of *KSS1* causes a reduction in agar penetration^26^, a phenotype called invasive growth that is related to filamentous growth^22^, while it has little effect on mating efficiency^27^. In contrast, deletion of *FUS3* allows cells to penetrate the agar more vigorously^26^ while they cause a moderate decrease in mating efficiency^27^. This and other data support the idea that one MAPK promotes invasive/filamentous growth (Kss1), and while another mainly functions to regulating mating (Fus3). Surprisingly, the elimination of both MAPKs results in more agar penetration, which identified an inhibitory role for the unphosphorylated form of Kss1 regulating filamentous growth^26–29^.

A p38-type pathway, the high osmolarity glycerol response (HOG) pathway, allows the response to hyperosmotic conditions through Pbs2 (MAPKK) and Hog1 (MAPK)^30–32^. One branch of this pathway shares components with the mating and fMAPK pathways^33^. Specifically, Ste20 and Ste11 function to regulate Pbs2 and Hog1. Therefore, MAPK pathways in yeast can share some common components despite the fact that the pathways induce different transcriptional and morphogenetic responses.

In pathogens, the filamentation response is critical for host-cell attachment, invasion into tissues, and virulence^34^. In *S. cerevisiae* haploid cells, filamentous growth is triggered by growth in a non-preferred carbon source. The response is regulated by multiple signal transduction pathways^35,36^, including the RAS-cAMP-PKA pathway^23,37–39^ and the filamentous growth MAPK pathway (fMAPK)^25^. These pathways induce target genes that reorganize cell polarity, the cell cycle, and cell adhesion to bring about a new cell type^40–43^. The signaling mucin Msb2 operates at the head of the fMAPK pathway, and through the adaptor protein, Sho1, regulates MAPK activity by interaction with the Ras-homology (Rho)-type GTPase Cdc42. Sho1 interacts with Msb2 and Ste11 and functions in both the fMAPK and HOG pathways^33,44,45^. Cdc42 is an essential gene that is required for the maintenance of cell polarity and signaling. Human homolog Cdc42 is 81% identical to the yeast protein^46–51^. Cdc42 regulates the fMAPK pathway by interacting with Ste20^25,26,40^.

Several mechanisms that promote insulation have been described. One mechanism involves scaffolds, such as Ste5^52–54^ and Pbs2^45^. Ste5 activates Fus3 by forming a multi-kinase complex that joins the Ste11, Ste7, and Fus3 kinases^52,55,56^. Pbs2 regulates the HOG pathway by being activated through two different branches, *SLN1-SSK1* and Sho1^45^. Another mechanism that is employed to maintain specificity involves cross-pathway inhibition. In this case, a transcription factor for the filamentation pathway, Tec1, is phosphorylated by Fus3, which leads to its turnover by a ubiquitin ligase complex^57,58^. An intriguing challenge, therefore, is to understand how pathways that share elements establish and maintain their identity^59,60^.

The core regulators of the fMAPK pathway (MAPKKK- > MAPKK- > MAPK) are well known, and several proteins have been identified that regulate the fMAPK pathway at or above the level of Cdc42. However, some proteins that regulate the fMAPK pathway may remain unidentified. For example, genome-wide screens have recently identified new proteins that regulate the fMAPK pathway^61^. Loss-of-function studies also have identified a broad set of genes that contribute to filamentous growth. Nevertheless, no single genetic approach can be expected to yield comprehensive results, and in this light, gene overexpression screens have proven to be an effective complement to gene deletion analysis^62,63^. Analysis of filamentation phenotypes from gene overexpression collections continues to provide a more comprehensive understanding of pseudohyphal growth regulation. We, therefore, performed an overexpression screen to identify new regulators of ERK-type pathways in yeast. Among the genes identified was a new pathway regulator, Mrs6, that when overexpressed stimulates the fMAPK pathway but not the HOG pathway. Since many of the new regulators identified have homologs in other eukaryotes, including humans, investigation of fMAPK pathway regulators provides a foundation for understanding MAPK pathway regulation in general. This may contribute to the development of new therapeutic targets in related species of fungal pathogens and can be linked to other signaling systems in higher organisms, with implications in the understanding and treatment of human disease.

## Results

### A genome-wide screen in yeast identifies new regulators of ERK-type MAPK pathways

**Figure 1:**
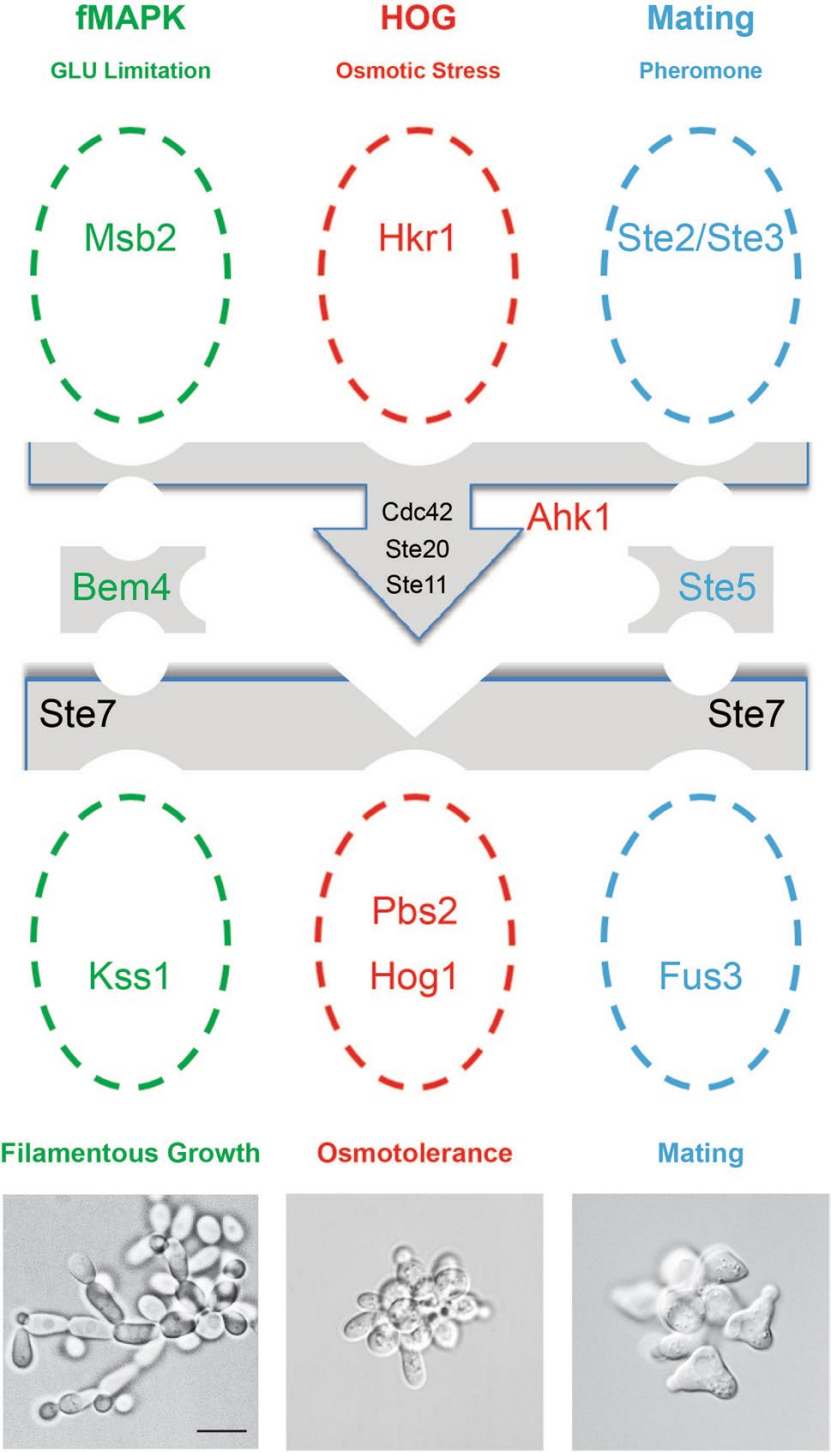
Three MAPK pathways in yeast share a subset of common components. Common components are shown in black, and pathway specific proteins are shown in color for the fMAPK (red), HOG (green), and mating (blue) pathways. Each pathway has a scaffold-type adaptor, Bem4^70^, Pbs2^45^ and Ahk1^71^, and Ste5^52^, and a specific MAP kinase. Cells undergo filamentous growth under nutrient-limiting conditions (left), cells do not change their morphology when exposed to YEP-GAL + 1.0 M KCl salt (middle), and YEP-GAL + 1 mg/ml α-factor stimulates an elongated cell shape or shmoo (right). Scale bar, 10 μm.

**Figure 2:**
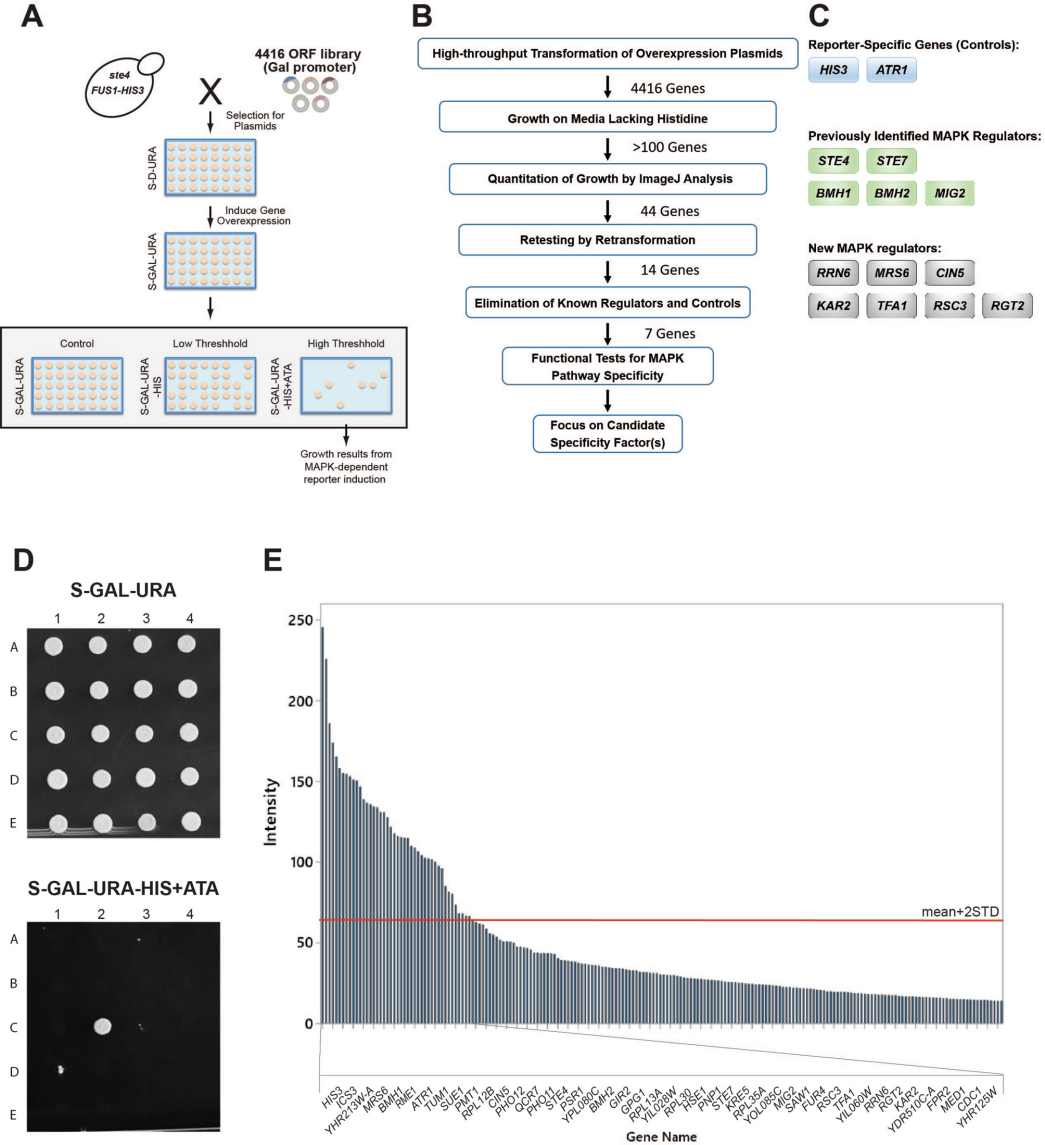
Genome-wide overexpression screen for new MAPK pathway regulatory proteins. **(A)** Diagram of the overexpression screen. An ordered collection of 4416 ORF overexpression plasmids covering ~ 80% of yeast genome controlled by an inducible (*pGAL1*) promoter (circles^66^) was introduced into a *ste4 FUS1-HIS3* strain (PC999) by high-throughput transformation. Transformants were generated by a microtiter plate method and pinned onto S-D-URA to select for plasmids. Overexpression of genes was accomplished by pinning colonies from S-D-URA to S-GAL-URA medium to induce overexpression of the genes. On the following day, cells were pinned to low threshold and high threshold (containing ATA, a competitive inhibitor of the His3 enzyme) media to identify genes that induce a MAPK pathway-dependent growth reporter (*FUS1-HIS3*) on media lacking histidine. The genes, which could overcome ATA, were identified as the candidates that, when overexpressed, can turn the pathway up (colored spots). **(B)** Pipeline for identifying functionally relevant MAPK pathway regulators. 44 genes were identified and prioritized for further analysis. The validation screen identified 14 genes from the initial screen. **(C)** The list of 14 genes that induced the MAPK pathway-dependent reporter, *FUS1-HIS3*, when overexpressed. Genes fell into three categories (see Table 1 for more details). **(D)** Example of a portion of one plate from the overexpression screen (the full screen is available in Table S4). The colony growing in the lower panel, C2, overexpresses *MRS6.* **(E)** The graph shows the results of the top genes identified by overexpression. Colony growth on S-GAL-URA-HIS + ATA resulting from reporter (*FUS1-HIS3*) expression was measured by ImageJ analysis. Growth based on spot intensity and determined and plotted in the graph. The top 200 genes are shown. Forty-four genes passed a cut-off of mean + 2STD (red bar) and are labelled here.

Three MAPK pathways in yeast require a subset of common components, including the Rho-type GTPase Cdc42, PAK Ste20, and MAPKKK Ste11, yet the pathways induce different responses [Fig. 1,^64,65^]. A genetic screen was performed to identify regulators of ERK-type MAPK pathways in yeast. An ordered collection of overexpression plasmids^66^ was examined for the induction of a MAPK pathway-dependent growth reporter [Fig. 2A, *FUS1-HIS3,*^67,68^]. The reporter provides a readout of two ERK-type MAPK pathways, mating and fMAPK (Fig. 1). However, cells lacking an intact mating pathway were evaluated (*ste4*Δ), which biases reporter activity towards the fMAPK pathway^69^.

Specifically, high-throughput transformation was used to introduce 4416 plasmids into a wild-type yeast strain (Fig. 2A)^69^ containing the *FUS1-HIS3* reporter. Gene overexpression was induced by galactose, as the plasmid library is designed to induce gene expression by the strong p*GAL*1 promoter. Galactose is also a non-preferred carbon source that stimulates filamentous growth. In this way, gene induction occurred under the same conditions that induce filamentous growth. Specifically, colonies were transferred in 96-well format from S-D-URA media to S-GAL-URA media to induce overexpression of the genes. After 24 h, cells were pinned from S-GAL-URA to S-GAL-URA (as a control for growth), S-GAL-URA-HIS, and S-GAL-URA-HIS containing 3-amino-1, 2, 4-triazole (ATA) media (10 µl of 2 M in 25 ml plates). ATA is a competitive inhibitor of the His3 enzyme, and its inclusion allows for selection for high levels of reporter activity ^72^. Genes that inhibited growth on S-GAL-URA-HIS (Fig. 2A, Low Threshold) may, when overexpressed, dampen reporter activity and will be discussed elsewhere. Genes that induce growth on S-GAL-URA-HIS + ATA media may stimulate MAPK pathway activity due to transcriptional up-regulation of the growth reporter (Fig. 2A). These genes, in principle, have the potential to encode new MAPK pathway regulatory proteins.

A scheme was employed to identify relevant MAPK pathway regulators, represented by a flowchart (Fig. 2B). In the initial screen, > 100 genes were identified that showed some growth on S-GAL-URA-HIS + ATA media (Fig. 2D, Table S4). To quantitatively assess differences in growth, colony size was measured by ImageJ analysis [Table S2, ^73^]. By applying a rigorous cut-off of the mean + 2SD, 44 genes were identified that showed elevated MAPK reporter activity when overexpressed (Fig. 2B,E). To independently validate genes identified by the screen, plasmids containing candidate genes were re-transformed into wild-type cells and re-tested for reporter activity. Fourteen genes passed this validation step (Fig. 2, B and C; see Table S3 for the raw data).

The genes that passed the above criteria fell into three categories (Fig. 2C, Table 1). The first category was metabolic controls. Two controls were identified, *HIS3*, which allows growth on media lacking histidine^74^, and *ATR1,* which encodes a multidrug efflux pump that confers ATA resistance^72^. The second category was known regulators of MAPK pathways. These included *STE4*, which regulates the mating pathway and complemented the signaling defect of the *ste4* mutant^75^; *STE7,* the MAPKK that regulates the mating and fMAPK pathways^76^; *BMH1* and *BMH2*, which are members of the 14-3-3 family of proteins and are established regulators of the fMAPK pathway ^7^, and *MIG2* a transcriptional repressor^77^ that has been implicated in fMAPK pathway regulation^78^. Not all components of the fMAPK pathway were identified: *STE20*, *STE50*, and *STE11* were not present in the collection; *MSB2* and *CDC42* would not be expected to be identified as C-terminal fusions of the proteins, which occur in the library, are not functional in the fMAPK pathway; *OPY2* was identified but fell below the threshold for statistical significance, and *TEC1* does not induce the growth reporter. In its unphosphorylated form, the MAPK Kss1 would also not be expected to activate the reporter and may not be identified for this reason^26,28,29^. *SHO1, BEM4,* and *STE12* were present in the collection but did not induce the reporter for reasons that have not been explored. The third category was potentially new MAPK pathway regulators. These included *RRN6*, *MRS6*, *CIN5*, *KAR2*, *TFA1*, *RSC3*, and *RGT2* (Fig. 2C, Table 1).

**Table 1:** Functional classification of MAPK pathway regulatory genes identified by gene overexpression alongside human homologs.

Genes	Standard name	Name description	Normalized growth intensity in WT^[a]^	Normalized growth intensity in *msb2*Δ^[a]^	Molecular Function^[b]^	**Biological Process**^**[b,c,d]**^	Human Homolog^[c]^
*YOR202W*	*HIS3*	HIStidine	22^[e]^	22	Allows cells to grow on media lacking histidine	Catalyzes the sixth step in histidine biosynthesis	NA
*YOR212W*	*STE4*	STErile	20	20	beta subunit of the first identified heterotrimeric G-protein	pheromone-dependent signal transduction involved in conjugation with cellular fusion, invasive growth in response to glucose limitation, regulation of transposition, RNA-mediated, chemotropism	G protein subunit beta 1(GNB1), GNB2, GNB5, GNB4, GNB3
*YDL159W*	*STE7*	STErile	18	13	MAP kinase kinase activity	MAPK cascade involved in cell wall organization or biogenesis, signal transduction involved in filamentous growth, invasive growth in response to glucose limitation, protein phosphorylation	mitogen-activated protein kinase kinase 2 (MAP2K2),MAP2K1
*YER177W*	*BMH1*	Brain Modulosignalin Homolog	18	9	RNA polymerase II activating transcription factor binding, DNA replication origin binding, phosphoserine binding	DNA damage checkpoint, signal transduction involved in filamentous growth, glycogen metabolic process,fungal-type cell wall chitin biosynthetic process, negative regulation of apoptotic process, pseudohyphal growth, negative regulation of transcription from RNA polymerase II promoter, Ras protein signal transduction	YWHAE, YWHAZ, YWHAB, SFN, YWHAG, YWHAH, YWHAQ
*YDR099W*	*BMH2*	Brain Modulosignalin Homolog	16	2	DNA replication origin binding, phosphoserine binding	DNA damage checkpoint, signal transduction involved in filamentous growth, glycogen metabolic process,fungal-type cell wall chitin biosynthetic process, negative regulation of apoptotic process, pseudohyphal growth, negative regulation of apoptotic process	YWHAE, YWHAB, SFN, YWHAG, YWHAH, YWHAQ
*YGL209W*	*MIG2*	Multicopy Inhibitor of Galactose gene expression	16	6	Zinc finger transcriptional repressor	cooperates with Mig1p in glucose-induced gene repression; under low glucose conditions relocalizes to mitochondrion, where it interacts with Ups1p, antagonizes mitochondrial fission factor Dnm1p, indicative of a role in mitochondrial fusion or regulating morphology; regulates filamentous growth in response to glucose depletion; activated in stochastic pulses of nuclear localization in response to low glucose	EGR1 ^[f]^
*YBL014C*	*RRN6*	Regulation of RNA polymerase I	16	2	RNA polymerase	Component of the core factor (CF) rDNA transcription factor complex; CF is required for transcription of 35S rRNA genes by RNA polymerase I and is composed of Rrn6p, Rrn7p, and Rrn11p	NA
*YOR370C*	*MRS6*	Mitochondrial RNA Splicing 5	15	2	Rab geranylgeranyltransferase activity, Rab GTPase binding	protein targeting to membrane, ER to Golgi vesicle-mediated transport, protein geranylgeranylation, activation of GTPase activity	CHM Rab escort protein (CHM), CHML
*YOR028C*	*CIN5*	Chromosome INstability	14	2	Basic leucine zipper (bZIP) transcription factor of the yAP-1 family	physically interacts with the Tup1-Cyc8 complex and recruits Tup1p to its targets; mediates pleiotropic drug resistance and salt tolerance; nuclearly localized under oxidative stress and sequestered in the cytoplasm by Lot6p under reducing conditions	NA
*YJL034W*	*KAR2*	KARyogamy	14	4	ATPase activity, unfolded protein binding	karyogamy involved in conjugation with cellular fusion, response to unfolded protein, SRP-dependent cotranslational protein targeting to membrane, translocation, fungal-type cell wall beta-glucan biosynthetic process	heat shock protein family A (Hsp70) member 5 (HSPA5)
*YKL028W*	*TFA1*	Transcription Factor a, subunit 1	13	< 0.1	TFIIE large subunit; RNA polymerase II core binding	involved in recruitment of RNA polymerase II to the promoter, activation of TFIIH, and promoter opening	general transcription factor IIE subunit 1 (GTF2E1)
*YDR303C*	*RSC3*	Remodel the Structure of Chromatin	13	< 0.1	Component of the RSC chromatin remodeling complex	essential gene required for maintenance of proper ploidy and regulation of ribosomal protein genes and the cell wall/stress response; RSC3 has a paralog, RSC30, that arose from the whole genome duplication	NA
*YDL138W*	*RGT2*	Restores Glucose Transport	12	< 0.1	Plasma membrane high glucose sensor that regulates glucose transport	low affinity sesnor that contains 12 predicted transmembrane segments and a long C-terminal tail required for hexose transporter induction; phosphorylation of the tail by Yck1p/Yck2p facilitates binding to the HXT co-repressors, Mth1p and Std1p; RGT2 has a paralog, SNF3, that arose from the whole genome duplication	solute carrier family 2 member 8 (SLC2A8), SLC2A10, SLC2A12
*YML116W*	*ATR1*	AminoTriazole Resistance	11	< 0.1	required for resistance to aminotriazole and 4-nitroquinoline-N-oxide	Multidrug efflux pump of the major facilitator superfamily; ATR1 has a paralog, YMR279C, that arose from the whole genome duplication; protein abundance increases in response to DNA replication stress	NA

^[a]^ Spot intensity was measured by ImageJ analysis and was normalized to wild-type values (see Table S2 for the raw data).

^[b]^ Data comes from SGD (https://www.yeastgenome.org/).

^[c]^ Data comes from Database Integration Tools (MARRVEL, Gene2Function, monarch INITIATIVE, ALLIANCE of GENOME RESOURCES, NCBI).

^[d]^ Not all biological processes are mentioned.

^[e]^ Growth intensity rates of the canididates validated in wild type is normalized to *msb2*Δ mutant.

^[f]^ Not all homologs are listed here.

To explore the characteristics of the genes identified by the screen, we used gene ontology (GO) annotations and database integration tools to identify the molecular and biological roles of proteins and determine whether they had mammalian homologs^79–84^. Many of the identified genes had human homologs with established functions in diverse biological processes (Table 1). These included *BMH1, BMH2* ^7^, *TFA1*^85^*, MRS6*^86,87^, and *KAR2*^88^. Moreover, the screen identified several essential genes (*MRS6*, *KAR2*, *TFA1*, and *RRN6*), and a set of paralogs (*BMH1* and *BMH2*), which might be missed in whole-genome deletion screens.

Many signaling pathways can influence the activity of the fMAPK pathway. One mechanism for this regulatory input comes from the regulation of the expression of the *MSB2* gene^89^. *MSB2* encodes the mucin-type glycoprotein that regulates the fMAPK pathway^69^. To determine whether these genes fall above or below Msb2 in their ability to stimulate MAPK pathway activity, candidates from the screen were examined for overexpression-dependent bypass the signaling defect of the *msb2*Δ mutant. A subset of the genes tested restored signaling in the *msb2*Δ mutant (Table 1, see Table S3 for the raw data), which indicates that they function below the level of Msb2 in the MAPK pathway. We were interested in new regulators that, when overexpressed, bypass the signaling defect of the *msb2*Δ mutant (*MRS6, RRN6,* and *KAR2*), because of their potential to modulate MAPK pathway activity directly.

### Examining the role of new MAPK pathway regulators in polarity reorganization during filamentous growth

**Figure 3:**
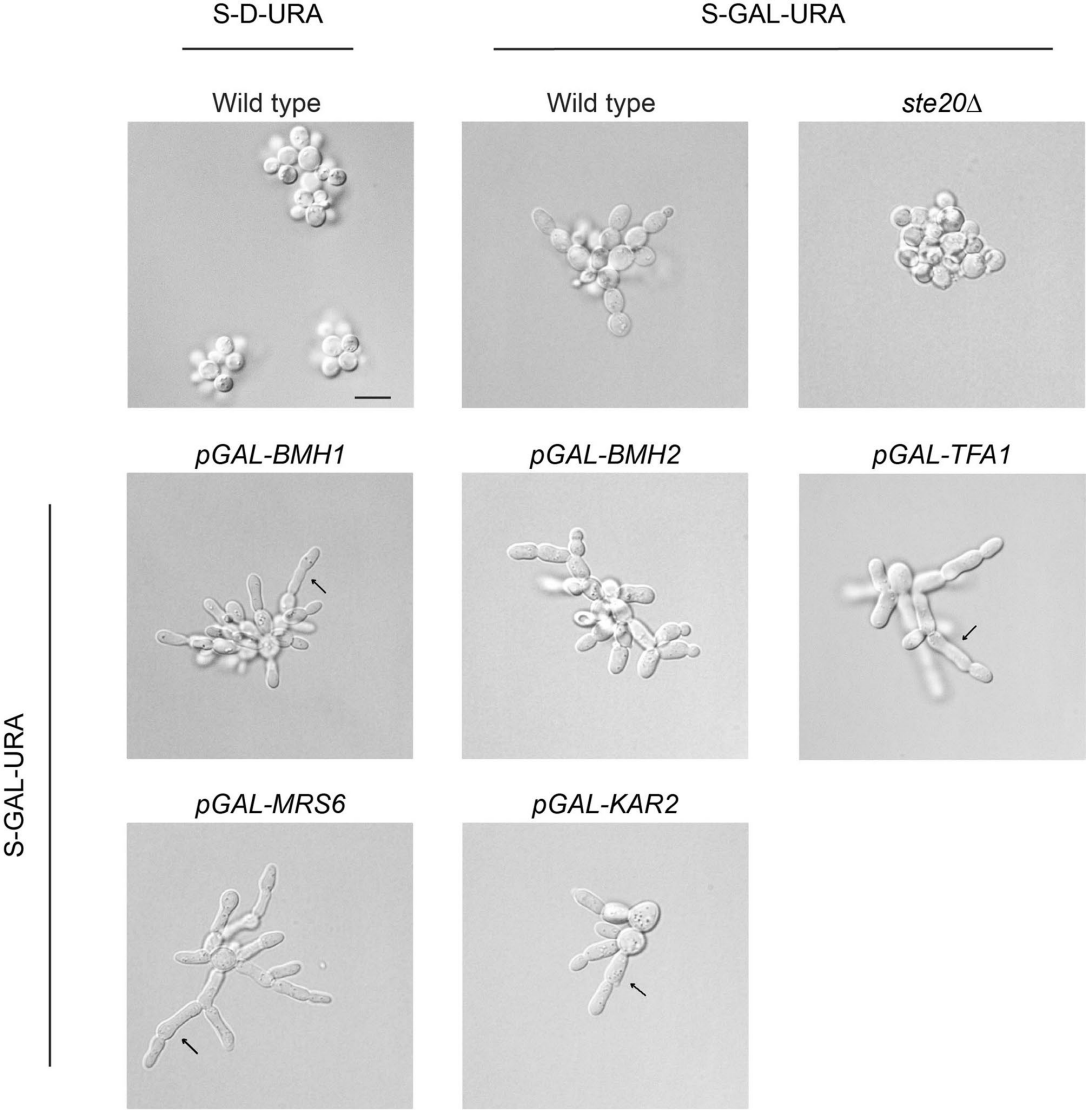
Morphological analysis of cells overexpressing genes that stimulate MAPK pathway signaling. (**A)** Cell morphology of the indicated strains by the single-cell invasive growth assay by DIC microscopy at 100X magnification. Scale bar, 10 μm. As controls, wild-type cells were grown in glucose (Glu, S-D) and galactose (S-Gal) media, and the *ste20*Δ mutant was grown in S-Gal media. Overexpression of *MRS6, BMH1, BMH2, KAR2,* and *TFA1* induced hyperpolarized morphologies. Arrows show elongated cells making chains of filaments.

During filamentous growth, yeast cells produce an elongated cell morphology, which results from hyper-polarized growth^90^. Hyperpolarized growth is caused by the fMAPK pathway^22^, which induces the expression of genes that cause a delay in the G1 and G2/M phases of the cell cycle^91,92^. The single-cell invasive growth assay^24^ was used to examine the polarized growth of a subset of candidate genes identified in the screen. Wild-type cells exist in the yeast form when grown in glucose (Fig. 3, S-D-URA) and undergo filamentous growth when grown in the non-preferred carbon source galactose (Fig. 3, S-GAL-URA). Cells lacking an intact fMAPK pathway are defective for filamentous growth by this assay (Fig. 3, *ste20*Δ). Overexpression of *MRS6, BMH1, BMH2, KAR2,* and *TFA1* induced hyperpolarized growth. Specifically, the cells were longer and had irregular morphologies (Fig. 3, arrows). This phenotype is distinct from activation of the HOG pathway, which shares components with the fMAPK pathway but does not induce a morphogenetic change when activated^21^.

### Analysis of fMAPK pathway regulatory proteins by functional tests for MAPK pathways

**Figure 4:**
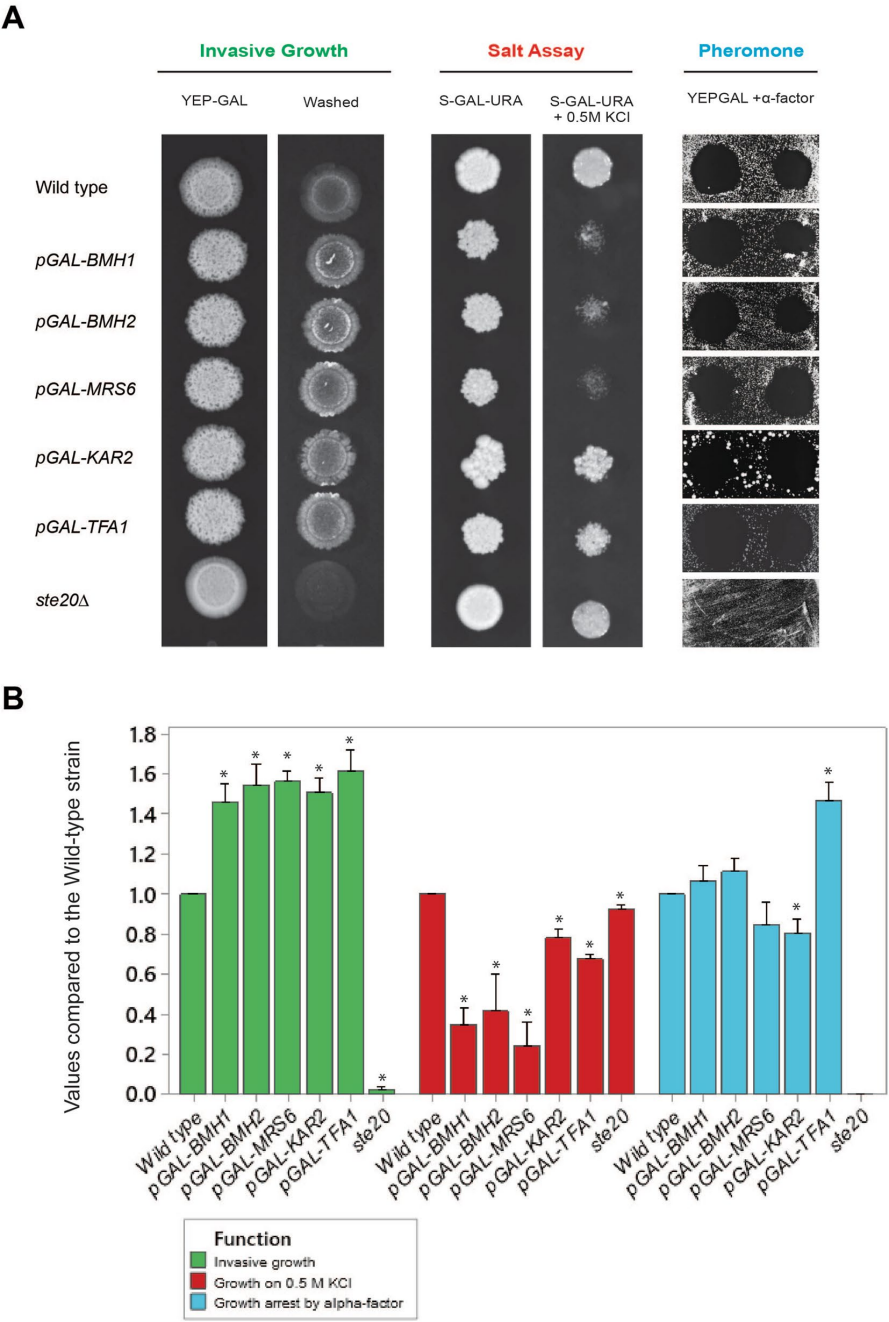
Phenotypic analysis of the role of overexpression of selected candidates on MAPK pathway activity. **(A)** Wild-type cells (PC6810) containing the indicated plasmids were grown in S-D-URA for 16 h and spotted onto the indicated media. For the PWA, cells were spotted onto YEP-GAL medium for 96 h. The plate was photographed (YEP-GAL), washed in a stream of water, and photographed again (Washed). To assess salt sensitivity, cells were spotted on S-GAL-URA and S-GAL-URA + 0.5 M KCl media for 72 h at 30 °C. To determine sensitivity to α-factor, cells were spread onto S-GAL-URA plates. 10 μl and 3 μl drops of 1 mg/ml α-factor were applied to the plates followed by incubation for 48 h. **(B)** Plot shows the quantified data for the invasive growth, salt assay, and pheromone. Values normalized to wild type (WT) values, which were set to a value of 1. Bars represent the average of at least three independent experiments. Error bars represent the standard deviation between trials. Asterisks indicate significant differences compared to the wild-type strain for the same condition (p-value < 0.01 by Student’s t-test).

Three MAPK pathways in yeast share components, including the Rho-type GTPase Cdc42, PAK Ste20, and MAPKKK Ste11 (Fig. 1, ^64,65^). As a complementary approach to assess the role of overexpression of *MRS6, BMH1, BMH2, KAR2,* and *TFA1* on these pathways, functional tests were performed that provide a readout of the three MAPK pathways. The plate-washing assay (PWA) measures the invasion of cells into the agar, which can be revealed by washing plates in a stream of water, and which is dependent on an intact fMAPK pathway^25^. Salt sensitivity was used to measure the activity of the HOG pathway^45,93^, and growth arrest by α-factor (halo assay) was used to measure the activity of the mating pathway^94^. Growth on galactose (YEP-GAL) resulted in hyper-invasive growth for each of the candidate genes tested by the PWA (Fig. 4A, green). Overexpression of *BMH1*, *BMH2*, and *MRS6* caused a growth defect on media containing salt (Fig. 4A, red). This result was interesting because the fMAPK pathway functions antagonistically with the HOG pathway ^95^. Thus, it is plausible that elevated activation of the fMAPK pathway by overexpression of these genes might result in a dampened HOG response. Overexpression of these genes did not result in a defect in halo formation (Fig. 4A, blue).

Quantifying the data also supports the idea that some genes turn fMAPK pathway up, and turn HOG down (Fig. 4B). These results support the idea that *MRS6, BMH1, BMH2, KAR2,* and *TFA1* stimulate the activity of the fMAPK pathway and might potentially play a specific role in that pathway when these genes are overexpressed.

### Mrs6 overexpression stimulates the fMAPK pathway and dampens the HOG pathway

**Figure 5:**
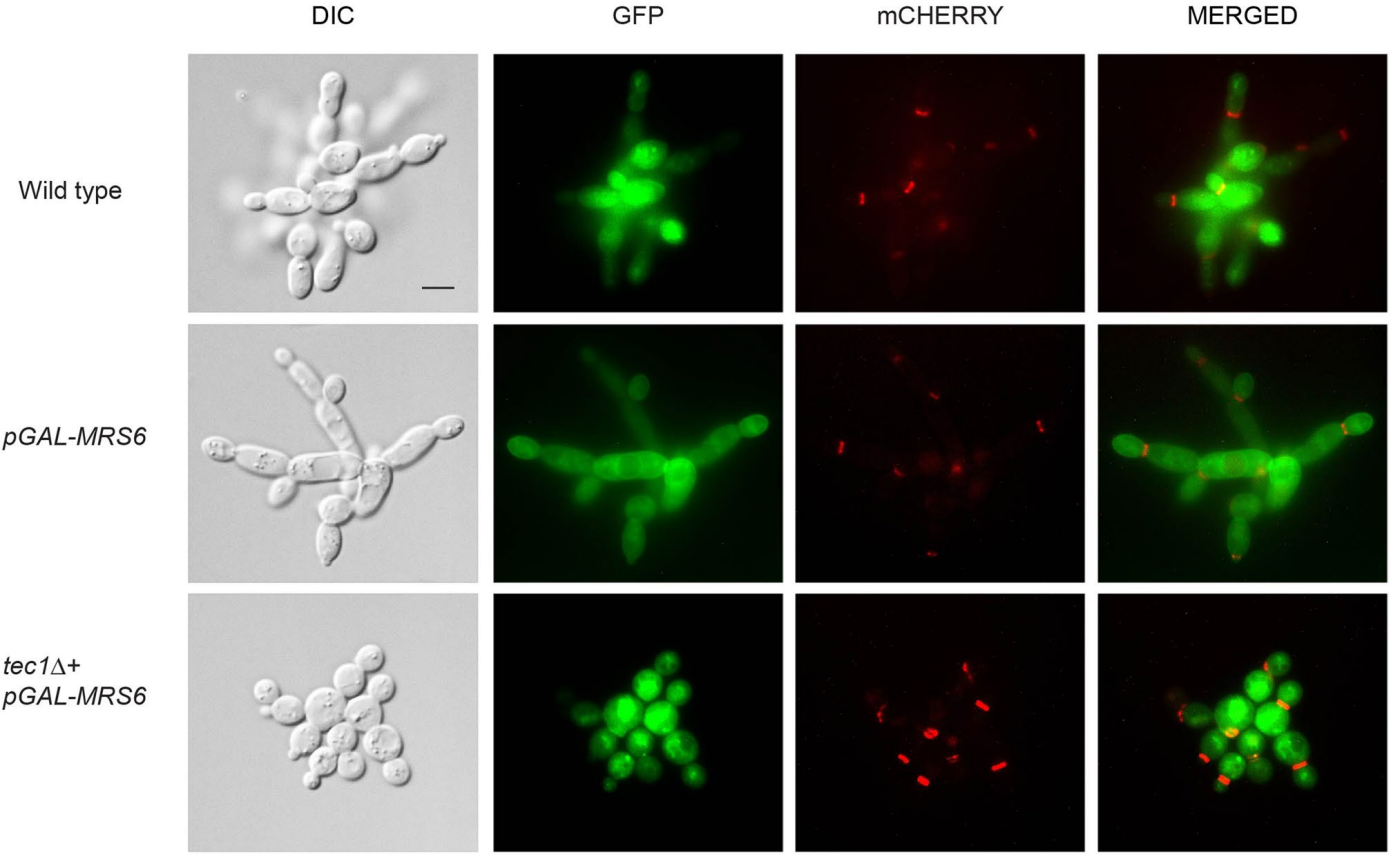
The localization of GFP-Cdc42 and the septin (by Cdc3-mCHERRY) in cells overexpressing *MRS6* with and without the transcription factor Tec1 were examined by fluorescence microscopy. Wild-type cells, and cells overexpressing *MRS6* in wild-type cells and the *tec1*Δ mutant cells were grown for 16 h in media [0.67% YNB without ammonium sulfate, 0.1% monosodium glutamate (MSG), 2% dextrose, 1 X amino acid stock without uracil, 0.36 mg/ml gent]. Cells were grown to mid-log phase for 6 h and photographed by fluorescence microscopy utilizing the GFP, Rhodamine, and DIC filter sets. Scale bar, 5 microns.

We focused on *MRS6* because it was one of the strongest hits from the screen (Fig. 2D, the spot represents *MRS6*). Overexpression of *MRS6* also strongly induced polarized growth (Fig. 3), induced hyper-invasive growth (Fig. 4, green), and dampened the HOG pathway (Fig. 4, red). Mrs6 is also an essential protein and, when overexpressed, bypassed the signaling defect of the *msb2* mutant (Table 1). Mrs6 is a Rab escort protein^96^ and has recently been identified as a modulator of the activity of the TOR pathway^97^. We confirmed that overexpression of *MRS6* induces hyperpolarized growth (Fig. 3). To determine whether this results from problems in cell polarity, we examined cells by fluorescence microscopy for defects in the localization of polarity proteins GFP-Cdc42 and septin Cdc3-mCHERRY ^98^. The localization of these proteins was normal in cells overexpressing *MRS6*, which indicates that Mrs6 does not promote cell elongation solely by perturbing proper cell morphogenesis*.* Interestingly, the elongated cell morphology seen in cells overexpressing *MRS6* was dependent on the fMAPK pathway, as it was not seen in the *tec1*Δ mutant, which lacks a key transcription factor for the pathway^40^ (Fig. 5). Additional examples of the morphology of these strains can be seen over a time-course experiment (Movies S1–S3). Taken together, these results provide support for a role for Mrs6 in positively regulating the fMAPK pathway.

**Figure 6:**
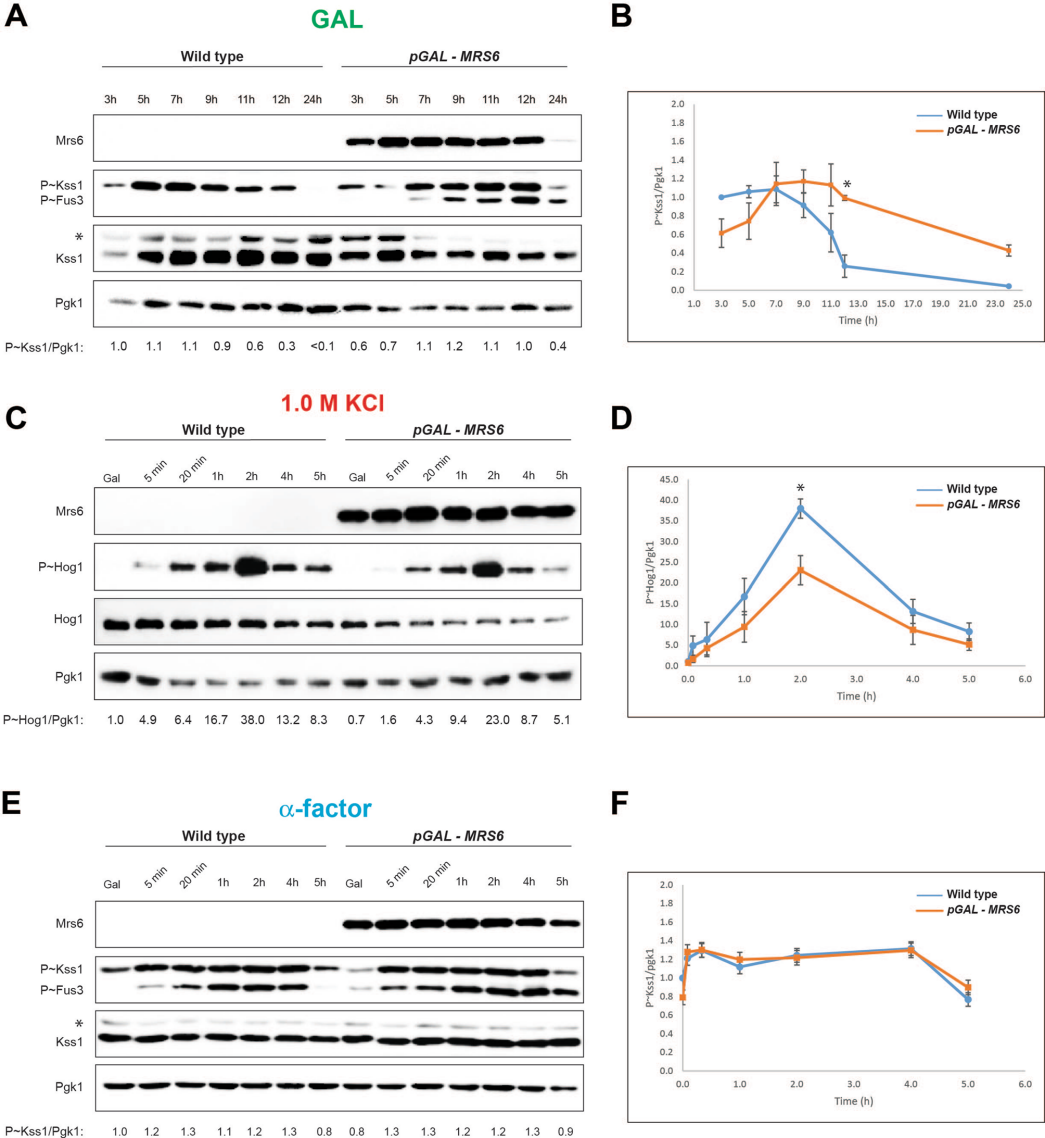
Impact of overexpression of *MRS6* on the fMAPK, HOG, and mating pathways. Wild-type cells (PC6810) and cells overexpressing *MRS6* (PC7447) were examined under conditions that induce MAPK pathway signaling. Cell extracts were evaluated by MAP kinase phosphorylation by immunoblot (IB) analysis. One example is shown for panels A, C, and E (raw data is shown in Fig. S3 A, B, and C, respectively). For panels B, D, and F, the data represent the average of at least three independent experiments. Error bars indicate the standard error of mean between trials (Asterisks, p-values < 0.05 by student’s t-test). (**A**) Cells were grown in the non-preferred carbon source galactose (YEP-GAL) for the times indicated. Cell extracts were examined by IB analysis for P ~ Kss1 and P ~ Fus3 by p44/42 antibodies. Mrs6 proteins were detected at ~ 91 kDa. Total Kss1 levels and Pgk1 (loading control, ~ 45 kDa) also were assessed. The ratio of P ~ Kss1 to Pgk1 normalized to wild-type values, which were set to a value of 1. (**B**) Graph visualizes the ratio of P ~ Kss1 to Pgk1 for wild-type and *pGAL-MRS6*. **(C)** Cells were pre-grown in YEP-GAL for 4 h following by growing in YEP-GAL medium containing 1.0 M KCl to examine P ~ Hog1. (**D**) Graph showing P ~ Hog1 to Pgk1 ratios, normalized to wild-type values, which were set to a value of 1. (**E**) Phosphorylation of Kss1 and Fus3 in response to pheromone. Cells were grown in YEP-GAL for 4 h, and incubated in YEP-GAL medium containing 1 mg/ml α-factor for the times indicated. Fus3 bands run in the same size as a degradation product of *MRS6*. (**F**) Graph showing P ~ Kss1 to Pgk1, normalized to wild-type values, which were set to a value of 1.

To explore the role of Mrs6 in regulating MAPK pathways, the phosphorylation of MAP kinases was examined, which provides a diagnostic readout of their activities. Based on immunoblot analysis, we typically see a > 100-fold increase in Mrs6 protein levels upon overexpression by the p*GAL1* promoter (Fig. 6A). This induction is similar to what has been reported for other proteins driven by that promoter ^66,99^. Using anti phospho p44-42 antibodies that detect the phosphorylated MAP kinases, Kss1 and Fus3, we found that overexpression of *MRS6* induced phosphorylation of Kss1 (Fig. 6A, P ~ Kss1). In comparison to wild-type cells, where the levels of P ~ Kss1 increased after 3 h growth in Gal and decreased after 7 h, overexpression of *MRS6* caused a delay in the phosphorylation of Kss1, which was sustained until 12 h and then decreased (Fig. 6B). This result indicates that *MRS6* alters the kinetics of the fMAPK pathway in a manner that might be expected to promote cell elongation during filamentous growth.

By comparison, the terminal MAP kinase in the HOG cascade, Hog1, was under-phosphorylated in response to salt due to *MRS6* overexpression (Fig. 6C). Immunoblot data indicated that overexpression of *MRS6* caused a modest reduction in HOG pathway activity (Fig. 6D). These results match with the fact that overexpression of *MRS6* caused a growth defect on high-osmolarity media (Fig. 4). Given that the fMAPK and HOG pathways can function antagonistically^95^, our results suggest that *MRS6* may be a specific regulator of the fMAPK pathway. Overexpression of *MRS6* did not have a dramatic effect on the mating pathway (Fig. 6, E and F). The main MAP kinase for the mating pathway, Fus3, is phosphorylated in response to pheromone. Although Fus3 phosphorylation was similar between wild-type cells and cells overexpressing *MRS6*, Fus3 migration overlapped with a degradation product of Mrs6 and was not used for quantitation. Therefore, *MRS6* overexpression led specifically to phosphorylation (activation) of the MAP kinase Kss1, which is consistent with a specific role for the protein in regulating the fMAPK pathway.

### Mrs6 interacts with the protein kinases Ste20 and Ste7

To define how Mrs6 might specifically regulate the fMAPK pathway, genetic suppression analysis was performed. Genetic suppression analysis can allow the ordering of proteins into a pathway using gain- and loss-of-function alleles. p*GAL-MRS6* was introduced into mutants that lack fMAPK pathway components. Reporter induction by overexpression of Mrs6 was compared in cells lacking components of the fMAPK pathway (Fig. 1). We looked at many components of fMAPK, including the *msb2*Δ, *sho1*Δ, *opy2*Δ, *ste20*Δ, *bem4*Δ, *ste50*Δ, and *ste11*Δ mutants. The results showed that Mrs6 overexpression partially bypassed the signaling defect of the *sho1*Δ mutant but not the *ste11*Δ mutant (*Fig. S1*, data shown for *sho1*Δ and *ste11*Δ). This experiment indicates that Mrs6 regulates the fMAPK pathway at or above the level of Ste11 in the fMAPK pathway. We also noticed that overexpression of *MRS6* induced a growth defect. The growth defect was separate from its induction of the fMAPK pathway, as it was seen in cells lacking fMAPK pathway components (*Fig. S2*). Interestingly, diploid strains heterozygous for *MRS6* also have a growth defect^100^.

**Figure 7:**
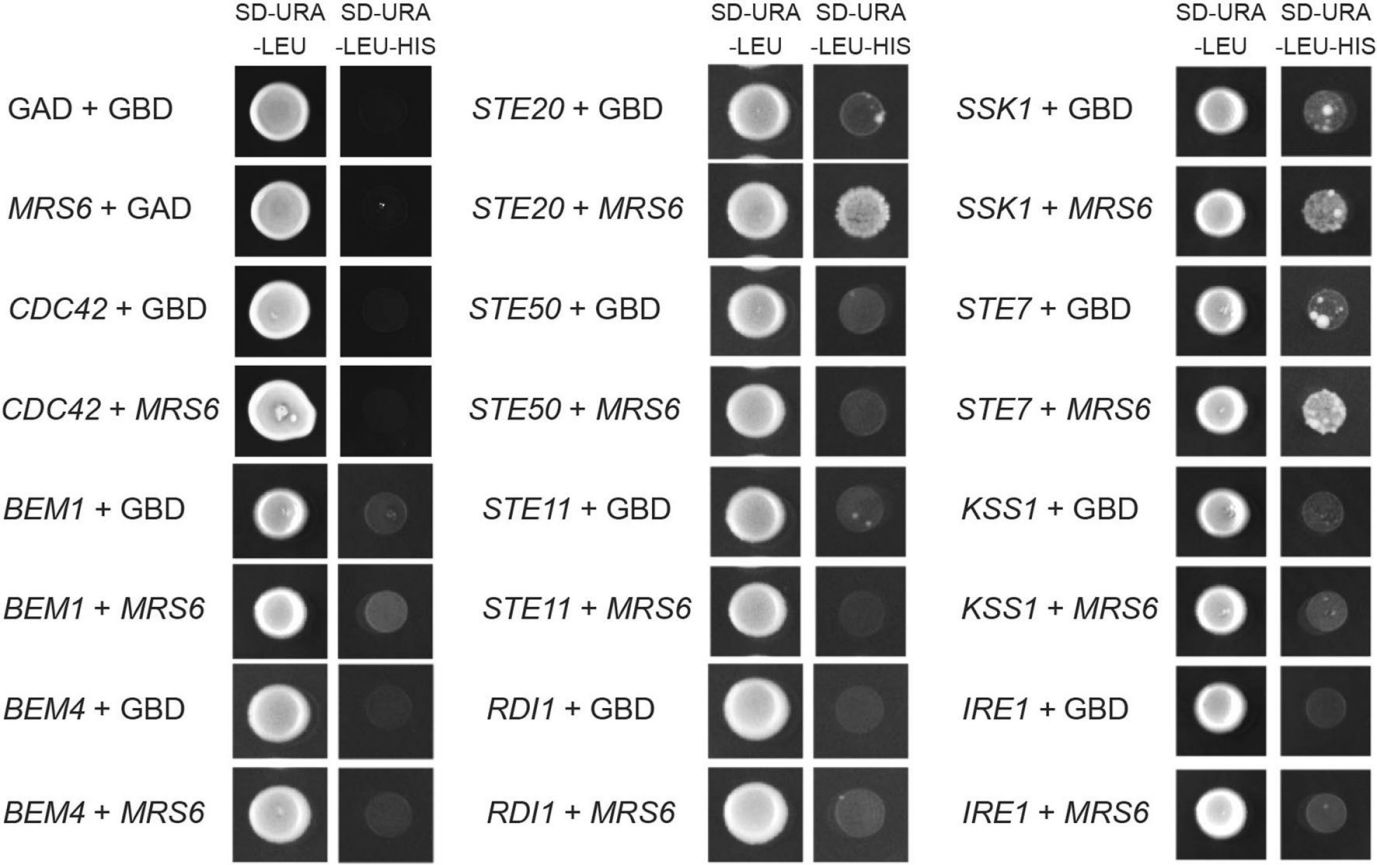
Two-hybrid analysis between Mrs6 and proteins that regulate fMAPK pathway. In the panels, GAD refers to pGAD-C1, and GBD refers to pGBDU-C1. Cells were grown on S-D-URA-LEU to maintain selection for the bait and prey plasmids. Growth on medium lacking histidine (S-D-URA-LEU-HIS) displayed an interaction of Mrs6 with MAPKKK kinase Ste20, and an interaction between Ste7 and Mrs6. Based on two-hybrid analysis, Mrs6 did not associate with other components of fMAPK.

To further define how Mrs6 regulates the fMAPK pathway, we analyzed the ability of Mrs6 to interact with fMAPK components by the two-hybrid system^101^. Two-hybrid analysis can identify protein interactions in vivo by reconstitution of the binding and activation domains of fusion proteins to the Gal4 transcription factor, evaluated by a growth reporter ^101^. Two-hybrid analysis has proven to be a useful tool in detecting interactions in many biological systems, including the isolated domains of interacting proteins^102,103^. The gene encoding Mrs6 was cloned into a two-hybrid vector (bait) and probed for interactions with a panel of proteins that regulate MAP kinase pathways. The analysis identified a robust interaction between Mrs6 and Ste20 (Fig. 7). Two-hybrid analysis also identified an interaction between Mrs6 and Ste7. Also, we saw a very weak positive signal for the Ssk1 protein. Mrs6 did not associate with other components of fMAPK by two-hybrid analysis. Therefore, the two-hybrid analysis may provide an explanation for how *MRS6* promotes fMAPK signaling, which includes the kinases Ste20, Ste11, Ste7, and Kss1, but not the HOG pathway, which includes the kinases Ste20, Ste11, Pbs2, and Hog1.

## Discussion

MAPK pathways regulate diverse cellular responses and are controlled by an expanding repertoire of regulatory proteins. In this study, we uncovered new regulators of an ERK-type MAPK pathway in yeast. We screened a S. cerevisiae library of covering 80% of the genome for genes that, when overexpressed, induce a MAPK-dependent growth reporter. Overexpression screens can uncover new roles for genes in biological processes and are well suited to identify roles for essential genes that cannot be evaluated by deletion analysis. In this study, we identified 12 regulatory genes of the MAPK pathway and two metabolic controls. The seven new genes identified in this study as MAPK pathway regulators provide a platform for exploring how a different cellular processes connect to and regulate MAPK pathways. We followed up on one of these candidates and showed by a combination of genetic and biochemical approaches that Mrs6 regulates the MAPK pathway that controls filamentous growth. The phenotypic difference between a wild-type strain and the indicated overexpression plasmids was apparent by reporter activity (Fig. 2), the elongated morphology of cells compared to wild-type cells (Fig. 3) and hyper-invasive growth by the plate-washing assay (Fig. 4A). These outcomes demonstrate that filamentous growth resulting from overexpression of these genes occurs under conditions (nutrient poor, galactose) when the pathway is active. Whether they also induce pathway activity under basal (nutrient-rich) conditions has not been tested. Furthermore, we found out Mrs6 interacts with kinases that regulate that pathway and might play a role in pathway specificity.

*MRS6* is an essential gene that may regulate the fMAPK pathway in several ways. One way might be through its role in regulating protein trafficking. Rab-type GTPases regulate protein trafficking^104–106^. Mrs6 is a Rab escort protein (REP) that makes a complex with the Rab GTPases Ypt1, Sec4, Ypt6, Vps21^107–109^. Given that Cdc42 is itself a component of the exocyst complex^110,111^, it is possible that Mrs6 might contribute to the delivery of Cdc42 or other fMAPK pathway components to the plasma membrane. Similarly, Mrs6 may regulate the assembly of the fMAPK signaling complex and/or its function in the secretory pathway. Ypt1 also regulates the UPR by promoting the decay of HAC1 RNA^112^. Interestingly, Msb2 and the fMAPK pathway are regulated by the UPR^113^. Perhaps some of the regulators identified in this study connect the MAPK pathway to the UPR pathway. In a related study, we found that *BMH1* and *BMH2* showed a connection to the UPR but not *MRS6* (Jamalzadeh et. al, unpublished data).

Mrs6 functions with members of the Rab family of GTPases, which control vesicle trafficking in the secretory pathway^114^. In particular, Mrs6 is a Rab-escort protein that promotes lipid modification (prenylation) of the Rab GTPase Ypt1 by the Bet2 and Bet4 geranylgeranyltransferase complex II^96^. Mrs6 specifically facilitates geranylgeranylation for the prenylation of Ypt1 at the Golgi through the Bet2 and Bet3 enzymes^115^. Mrs6 might regulate the fMAPK pathway through its role in regulating Rab prenylation. Ypt1 and the Bet proteins are essential proteins that cannot be readily analyzed by deletion analysis. We found that Bet proteins, Bet3, and the Rab GTPase, Ypt1, when overexpressed did not impact fMAPK pathway activity (Table S4, see labeled genes). Mrs6 has also been identified as a modulator of the TOR pathway by interacting with the transcription factor Sfp1^97,116^. Sfp1, when overexpressed, did not impact the activity of the fMAPK pathway (Table S4, see labeled genes). However, interestingly, overexpression of *SFP1* stimulates filamentous growth^117^. Thus, Mrs6 may have a separate function in regulating the fMAPK pathway than its role in Rab or TOR pathway regulation. In the fMAPK pathway, the Rho-type GTPase Cdc42 is modified by lipid geranyl groups (by Cdc43); thus, Mrs6 may impact the lipid modification of Cdc42. However, Mrs6 did not associate with Cdc42 by two-hybrid analysis.

Two-hybrid analysis showed that Mrs6 interacts with Ste20. Ste20 is the PAK kinase that regulates the fMAPK pathway^25,118,119^. Ste20 is recruited by a complex containing Cdc42 and Cdc24 to the membrane^120^. Thus, Mrs6 may regulate the fMAPK pathway by promoting the plamsa membrane recruitment or activation of Ste20. Mrs6 also interacts with Ste7 (a MAPKK). Given that *MRS6* specifically stimulates the fMAPK pathway, Mrs6 might facilitate interactions among members of the kinase cascade. In support of this possibility, overexpression of *MRS6* dampened the activity of the HOG pathway. Alternatively, Mrs6 may interact with Ste20 in one complex and Ste7 in another complex. Future studies will be required to determine how these interactions promote fMAPK pathway induction.

Signal transduction pathways operate in different ways with vastly different kinetics^5^. The activation kinetics of signaling pathways are crucial to determine the nature of the biological response. The fMAPK pathway operates with slower kinetics compared to the mating and HOG pathways [^121^, this study]. The kinetics of activation has probably been fine-tuned for the filamentous growth response. Overexpression of *MRS6* increases fMAPK pathway activity (see Fig. 6B). By examining the kinetics of the fMAPK pathway, we show that overexpression of *MRS6* extends the amount of time the pathway is active. The interactions between Mrs6 with Ste20 and Ste7 might extend pathway activity. Phenotypically, this may augment the MAPK-dependent cell-cycle delays, resulting in hyper-invasive growth, which we also observe upon *MRS6* overexpression.

In a separate study, *MRS6* was shown to regulate the TORC1 pathway through *SFP1* to control ribosome biogenesis^97,116^. TOR is a master regulatory pathway of cell growth and nutrient sensing^122^. TOR’s activator, GOLPH3, has been identified recently as an oncogene in many human cancers^123^. Hence, the identification of Mrs6 as a key regulator of the fMAPK pathway in yeast raises the possibility that REP1/REP2 may link fMAPK signaling to the TOR pathway and to the secretory system in higher organisms.

In mammalian cells, *MRS6* homolog encoded by CHM, which is the human Rab escort proteins REP1/CHM or REP2/CHML and share 50% sequence identity with Mrs6^86^. CHM is a disease of the retina, which causes progressive vision loss^12^. Furthermore, REP1/CHM has been shown to regulate the epidermal growth factor receptor (EGFR) through the transcription factor STAT3. EGRF is also a major regulator of the Grb-SOS-RAS-MEK-ERK pathway, which is commonly misregulated in cancer cells ^124^. Given that EGFR also signals through RAS-MEK-ERK^125,126^, our screen may have identified a new and general regulator of ERK-type MAPK pathways.

## Materials and methods

### Strains and plasmids

Strains used in the study are listed in Table S1. Strains were cultured in yeast extract and peptone (YEP) media (1% yeast extract and 2% bactopeptone) with a source of carbon [2% glucose (D) or 2% galactose (GAL)] for growth in liquid culture or 2% agar for growth in semi-solid agar media. All experiments were carried out at 30 °C unless otherwise specified. Synthetic complete (S) medium was used for maintaining selection for plasmids. Bacterial cultures of *Escherichia coli* were proliferated in LB + CARB media (carbenicillin) by standard methods ^127^. The pRS plasmids (pRS315 and pRS316) have been described ^128^. To construct two-hybrid plasmids, plasmids pGAD-C1 and pGBDU-C1 were used ^129^.

### Analysis of a gene overexpression collection for altered activity of a MAPK pathway-dependent growth reporter

A microtiter-based high throughput transformation method^130^ was used to introduce a collection of ~ 4500 plasmids, each overexpressing a different yeast gene^66^ into strain (PC999). Transformants were screened for Msb2-HA secretion as described^89^ and the activity of the fMAPK pathway in this study. Specifically, transformants were pinned onto S-D-URA to select for plasmids. Colonies that grew onto S-D-URA were then pinned to S-GAL-URA to induce gene overexpression. From S-GAL-URA, cells were pinned onto S-GAL-URA, S-GAL-URA-HIS, and S-GAL-URA-HIS + ATA to identify positive regulators of the fMAPK pathway. Colonies that grew on S-GAL-URA-HIS + ATA media resulted from elevated fMAPK pathway activity due to the up-regulation of the growth reporter (*FUS1-HIS3*).

### Genome-wide screen and data analysis

The growth of 4416 genes was examined from 46 plates (raw data is available in Table S4, see labeled genes for hits). Not all of the genes from the collection were analyzed. This may have resulted because of the failure of some plasmids to be transformed and contamination on several plates. ImageJ analysis (https://imagej.nih.gov/ij/) was used to quantify colony growth. Images of the plates from the screen were converted to 8-bit and inverted. A threshold adjustment was performed, followed by analysis by the DNA microarray plugin to measure spot intensity for each colony (Table S2). Outputs from ImageJ were saved as cvs format for additional analysis.

A MATLAB script was written to identify growth that was statistically significant. A cut-off of mean + 2STD identified the top 3% of genes that, when overexpressed, showed growth that was above background. Validation of candidates was performed by re-transformation of plasmids containing genes, by standard transformations ^131^, into a wild-type strain (PC6021) and testing for reporter induction by growth on S-GAL-URA-HIS + ATA media (Table S3). The same plasmids were also transformed into the *msb2*Δ mutant (PC3209) to determine the bypass of that regulator of the pathway (Table S3). Database Integration Tools were used for further describing the identified genes’ characteristics and their orthologs in a concise manner^79–84^.

### Microscopy

Differential interference contrast (DIC) microscopy was performed at 100X using an Axioplan 2 fluorescent microscope (Zeiss) with a Plan-Apochromat 100X/1.4 (oil) objective (N.A. 1.4) (coverslip 0.17) (Zeiss). Digital images were obtained with the Axiocam MRm camera (Zeiss) and Axiovision 4.4 software (Zeiss) was used for image acquisition. Adobe Photoshop was used for brightness and contrast adjustments. Polarized cells were assigned by examining cells over multiple focal planes by DIC.

### Localization and fluorescence microscopy

Wild-type cells with integrated Cdc3-mCherry that also contained pGFP-Cdc42 and either p*RS316* (PC7589) or p*GAL-MRS6* (PC7590), and *tec1*Δ cells with integrated Cdc3-mCherry and contained pGFP-Cdc42 and p*GAL-MRS6* (PC7592) were examined by fluorescence microscopy. Plasmids were selected on S-D-URA + Geneticin (Cat#11811-031) semi-sold agar media [2% agar, 0.67% YNB without ammonium sulfate, 0.1% monosodium glutamate (MSG), 2% dextrose, 1 X amino acid stock without uracil, 0.36 mg/ml Gent]^132^ at 30^◦^C. Samples were grown for 16 h in S-D-URA + 0.1% MSG + 0.36 mg/ml Gent. Five hundred microliters of each culture was collected by centrifugation, washed twice in distilled water and transferred to 10 ml of YEP-GAL + MSG + Gent media. Cells were grown for 6 h. The mid-log phase samples were washed twice with water, and cells were examined by fluorescence microscopy at 100X utilizing GFP, Rhodamine, and DIC filter sets using an Axioplan 2 fluorescent microscope (Zeiss). Cells were examined at serial sections on the plane of the Z-axis. Brightness and contrast were adjusted to reduce background using Adobe Photoshop.

Time-lapse fluorescence microscopy was performed as described^132^. Cells were grown for 16 h in in S-D-URA + 0.1% MSG + 0.36 mg/ml Gent at 30 °C. Approximately 800 µl of YEP-GAL + 1% agarose was placed on 12 mm Nunc glass base dishes (150,680, Thermo Scientific, Waltham, MA). One thousand microliters of cells were washed in water and resuspended in YEP-GAL media. 25 µl of cells were placed underneath of the agarose pad by gently lifting the pad with a scalpel. The plate was incubated for 30 min at 30^◦^C for stabilizing the cells. Cells were examined by a Zeiss 710 confocal microscope equipped with a Plan-Apochromat 40x/1.4 Oil DIC M27 for 5 h with 10 min intervals. Serial sections were examined in the plane of the Z-axis.

### Functional assays for MAPK pathway activity

Cell morphology was assessed by the single-cell invasive growth assay ^24^. Invasive growth was assessed by the PWA ^25^. For the PWA, equal concentrations of cells were spotted onto YEP-GAL media. The activity of the HOG pathway was assessed by growth on high-osmolarity media. Equal concentrations of cells were spotted onto S-GAL-URA and S-GAL-URA + 0.5 M KCl media. Halo assays were performed as described ^133^. Cells were spotted onto YEP-GAL media followed by spotting 3 μl and 10 μl α-factor (1 mg/ml) on the plate. Plates were incubated at 30 °C and photographed at 24 h and 48 h. The single-cell invasive growth assay was performed as described ^24^.

### Phospho-immunoblot analysis

Phosphorylation of different MAP kinases in response to different stimuli was examined as described ^134,135^. Cells were grown to mid-log phase from a saturated culture in YEP-D or YEP-GAL media for 4 h. Cells were washed and sub-cultured into YEP-GAL, YEP-GAL with 1.0 M KCl, or YEP-GAL with α-factor (1 mg/ml). Cells were collected at various times by centrifugation, washed once, and stored at -80 ˚C. Proteins were extracted by trichloroacetic acid precipitation (TCA) and resuspended in 0.15 ml sample buffer by heating to 90 °C. Protein samples were separated by sodium dodecyl sulfate–polyacrylamide gel electrophoresis (SDS-PAGE) (10% acrylamide). Proteins were transferred from polyacrylamide to nitrocellulose membranes (AmershamTM ProtranTM Premium 0.45 μm NC, GE Healthcare Life sciences, 10600003) by electrotransfer (Bio-Rad laboratories Inc.). Membranes were blocked with 5% BSA in 1X TBST (10 mM TRIS–HCl pH 8, 150 mM NaCl, 0.05% Tween 20).

Phosphorylation of mating and fMAPK pathways (P ~ Kss1 and P ~ Fus3) was investigated with p44-42 antibody (Cell Signaling Technology, Danvers, MA, 4370) at a dilution of 1:10,000 to detect ERK-type MAP kinases. Phosphorylated Hog1 was detected using a 1:10,000 dilution of α-phospho p38 antibody (Santa Cruz Biotechnology, Santa Cruz CA; #yC-20). Total Kss1 was detected with α-Kss1 antibodies (Santa Cruz Biotechnology, Santa Cruz, CA; #6775) at a 1:5,000 dilution. Total Hog1 was detected with α-Hog1 antibodies at a 1:5,000 dilution and Pgk1 was detected using mouse monoclonal antibodies at a 1:5,000 dilution (Novex, 459250). Membranes were incubated 16 h with primary antibodies in 1X TBST with 5% BSA at 4 °C. Control membranes were incubated 16 h in Pgk1 antibodies in 1X TBST with 5% non-fat dried milk at 4 °C. To detect the primary antibodies, secondary antibodies of goat anti-rabbit IgG-HRP at a 1:10,000 dilution (Jackson ImmunoResearch Laboratories, Inc., West Grove, PA, 111–035-144), and Goat α-mouse secondary (Bio-Rad Laboratories, Hercules, CA, 1706516) at a 1:5,000 dilution were used within milk as blocking buffer. The *pGAL-MRS6* plasmid encodes a Mrs6-HA-HIS-Protein A fusion protein, which can be detected with the abovementioned antibodies. Blots were visualized by chemiluminescence using a Bio-Rad ChemiDoc XRS + system (Bio-Rad, 1708265). Image Lab Software (Bio-Rad, Inc.) was applied to analyze the band intensity.

### Genetic suppression analysis

Control (pRS316) and *pGAL-MRS6* plasmids were transformed into wild-type strain (PC538) and MAPK pathway mutants. These included *msb2*Δ (PC3209), *sho1*Δ (PC5692), *opy2*Δ (PC3752), *ste20*Δ (PC5692), *bem4*Δ (PC3551), *ste50*Δ (PC610), and *ste11*Δ (PC3861) mutants. Cells were grown on S-GAL-URA and S-GAL-URA-HIS to evaluate growth, which we infer to represent bypass of the mutant phenotype.

### Cloning the MRS6 gene into two-hybrid plasmids

The *MRS6* gene was cloned into the pGAD-C1 and pGBDU-C1 vectors in the following way. The *MRS6* gene was amplified by PCR using the forward primer 5′-ATGCATCGATATGTTAAGTCCTGAACGTAGACC-3′ and reverse primer 5′-ATGCGTCGACTCATATCTCCATTTCACCTACAAATTC-3′. The PCR product was purified with QIAquick PCR Purification Kit, Qiagen, CA#28106. The PCR product and pGAD-C1 vector were digested with ClaI (5′-ATCGAT-3′, New England BioLabs Inc., MA, CA#R0197S) and SalI (5′-GTCGAC-3′, New England Biolabs Inc., MA, CA#R3138S) restriction enzymes. Digested insert and vector DNAs were run on a 1% agarose gel containing ethidium bromide. Bands were extracted from the gel using the QIAquick Gel Extraction Kit, Qiagen (CA#28704). A quick Ligation Kit (New England Biolabs Inc., MA, CA#M200l) was used for ligating the insert and vector. The ligation mixture was transformed into *E. coli* (One-Shot MAX Efficiency DH5α-T1 Competent Cells, ThermoFisher, CA# 12297016), followed by plating on LB + Carb plates. The plates were incubated at 37 °C for 24 h. Transformants were confirmed by digestion with ClaI and SalI. Plasmids were sequenced at the Roswell Park Sequencing facility (Roswell Park Cancer Institute, Buffalo, NY).

### Two-hybrid assay

Two-hybrid constructs (pGBDU-C1 bait and pGAD-C1 prey) and empty vectors as controls were introduced into strain PJ694A (PC284)^129^ using the lithium acetate transformation standard protocols^136^. Transformants were selected on S-D media lacking uracil (URA) and leucine (LEU) to maintain selection for plasmids. Protein–protein interactions were screened by spotting cells onto S-D-URA-LEU media that was also lacking histidine (HIS) and containing ATA. Growth in this media results from the induction of a two-hybrid transcriptional reporter as the readout of protein–protein interactions.

## Supplementary information

Supplementary Information.

Supplementary Table s1.

Supplementary Table s2.

Supplementary Table s3.

Supplementary Table s4.

Supplementary Figure s1.

Supplementary Figure s2.

Supplementary Figure s3.

Supplementary Figure s4.

Supplementary Figure s5.

Supplementary Video.

Supplementary Video.

Supplementary Video.

## Abbreviations

ATA: 3-Amino-1, 2, 4-triazole
CARB: Carbenicillin
CHM: Choroideremia
CHML: Choroideremia-like
D: Dextrose
DIC: Differential interference contrast
*E. coli*: *Escherichia coli*
ERK: Extracellular-signal-regulated kinase
GAL: Galactose
GLU: Glucose
GAD: Gal4 activation domain
GBD: Gal4 binding domain
Gent: Geneticin
GO: Gene ontology
GTP: Guanine nucleotide triphosphate
HIS: Histidine
HOG: High osmolarity glycerol response
LEU: Leucine
MAPK: Mitogen-activated protein kinase
MAPKKK: Mitogen-activated protein kinase kinase kinase
MEKK: Mitogen-activated protein kinase kinase kinase
MEK: Mitogen-activated protein kinase kinase
MSG: Monosodium glutamate
PAK: P21-activated protein kinase
PWA: Plate washing assay
RabGDI: RabGDPdissociation inhibitor
RabGGTase: Rab geranylgeranyl transferase
REP: Rab escort protein
Rho: Ras homology
SDS-PAGE: Sodium dodecyl sulfate–polyacrylamide gel electrophoresis
STD: Standard deviation
S: Synthetic
TCA: Trichloroacetic acid
URA: Uracil
WT: Wild type
YEP: Yeast extract and peptone
