# Supplementary material for: A Rab escort protein regulates the MAPK pathway that controls filamentous growth in yeast
Source: Sci Rep. 2020 Dec 17;10:22184. doi: 10.1038/s41598-020-78470-4 (PMC7746766; doi:10.1038/s41598-020-78470-4)

Supplementary Materials

A Rab Escort Protein Regulates the MAPK Pathway That Controls Filamentous Growth in Yeast

Sheida Jamalzadeh [1], Atindra N. Pujari [2], Paul J. Cullen [2] †

1. Department of Chemical and Biological Engineering, University at Buffalo, State University of New York, Buffalo New York

2. Department of Biological Sciences, University at Buffalo, State University of New York, Buffalo New York

† Corresponding author: Paul J. Cullen
Address: Department of Biological Sciences
532 Cooke Hall
State University of New York at Buffalo
Buffalo, NY 14260-1300
Phone: (716)-645-4923
FAX: (716)-645-2975
E-mail: pjcullen@buffalo.edu

Figure S1. Growth of the indicated strains on S-GAL-URA and S-GAL-URA-HIS media. Serial dilutions were spotted.

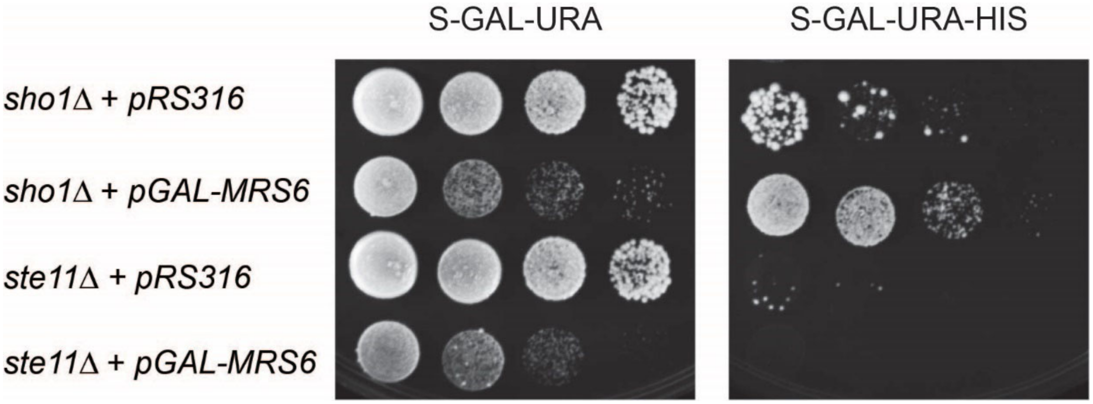

Figure S2. Growth of the indicated strains on S-D-URA, S-GAL-URA and S-GAL-URA+1M KCl media.

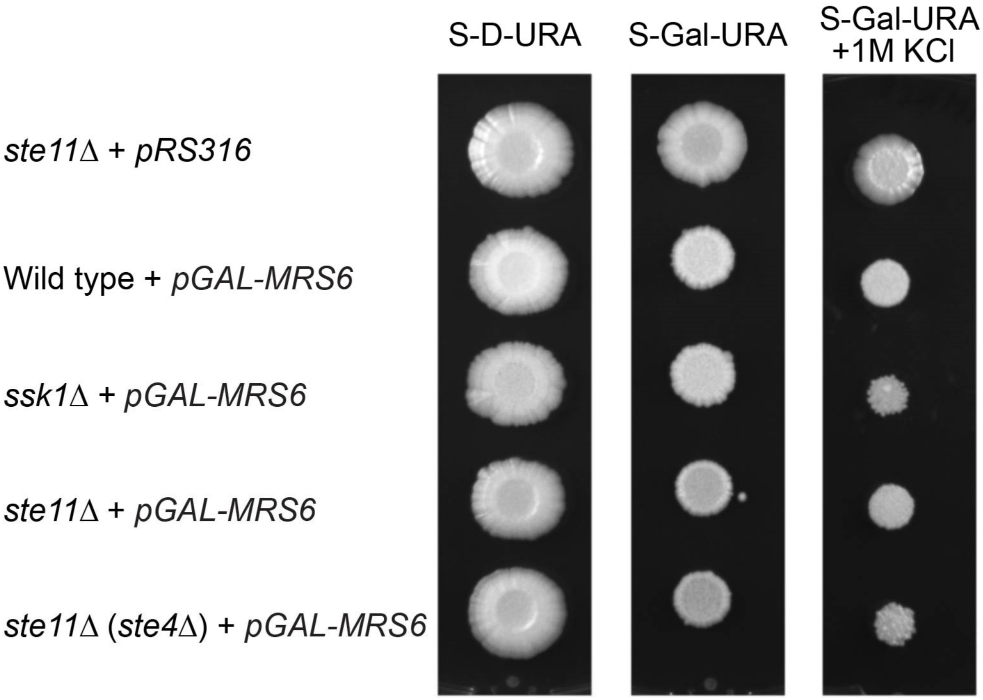

Figure S3A. Raw material for Fig. 6A.

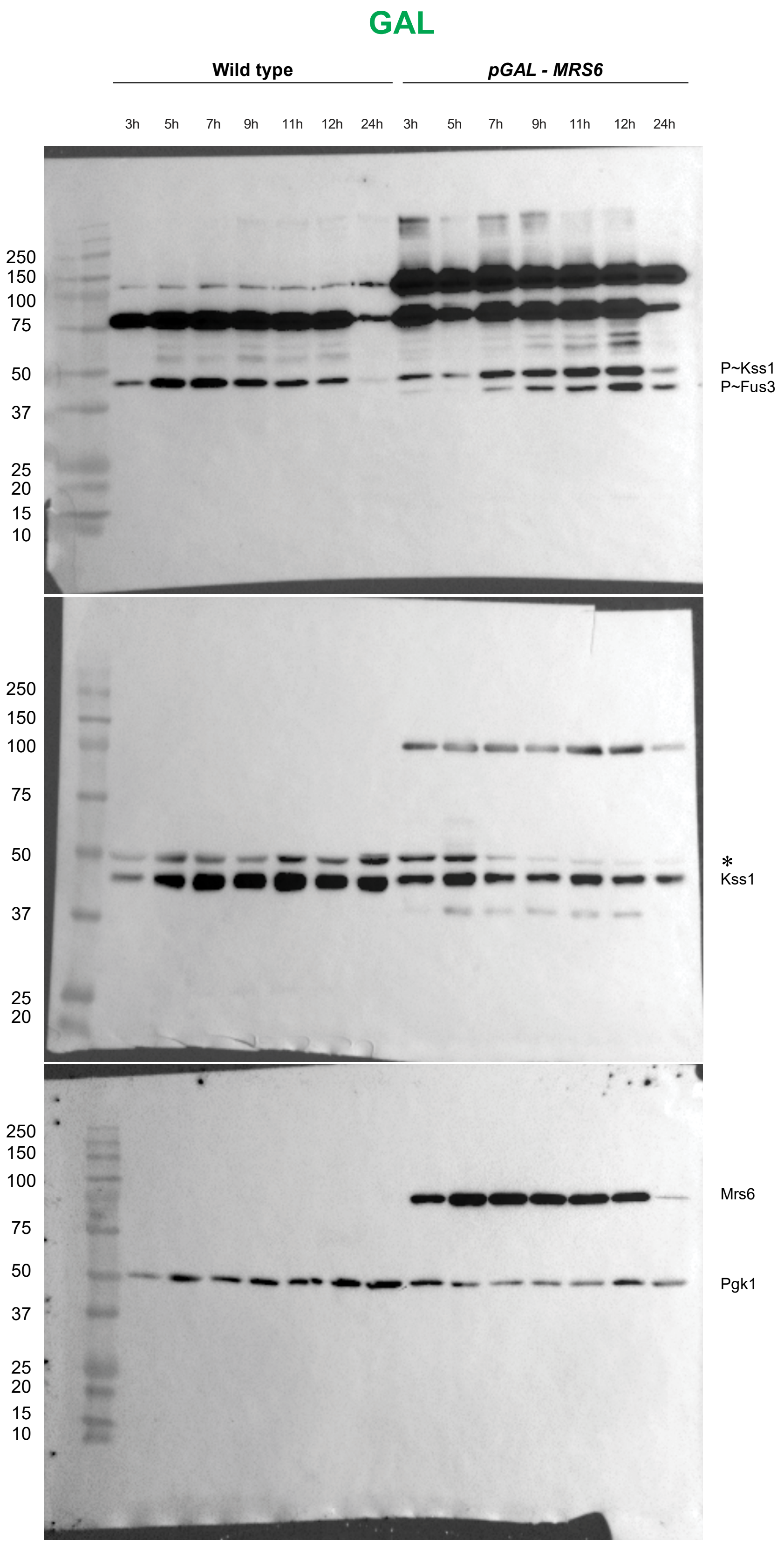

Figure S3B. Raw material for Fig. 6C.

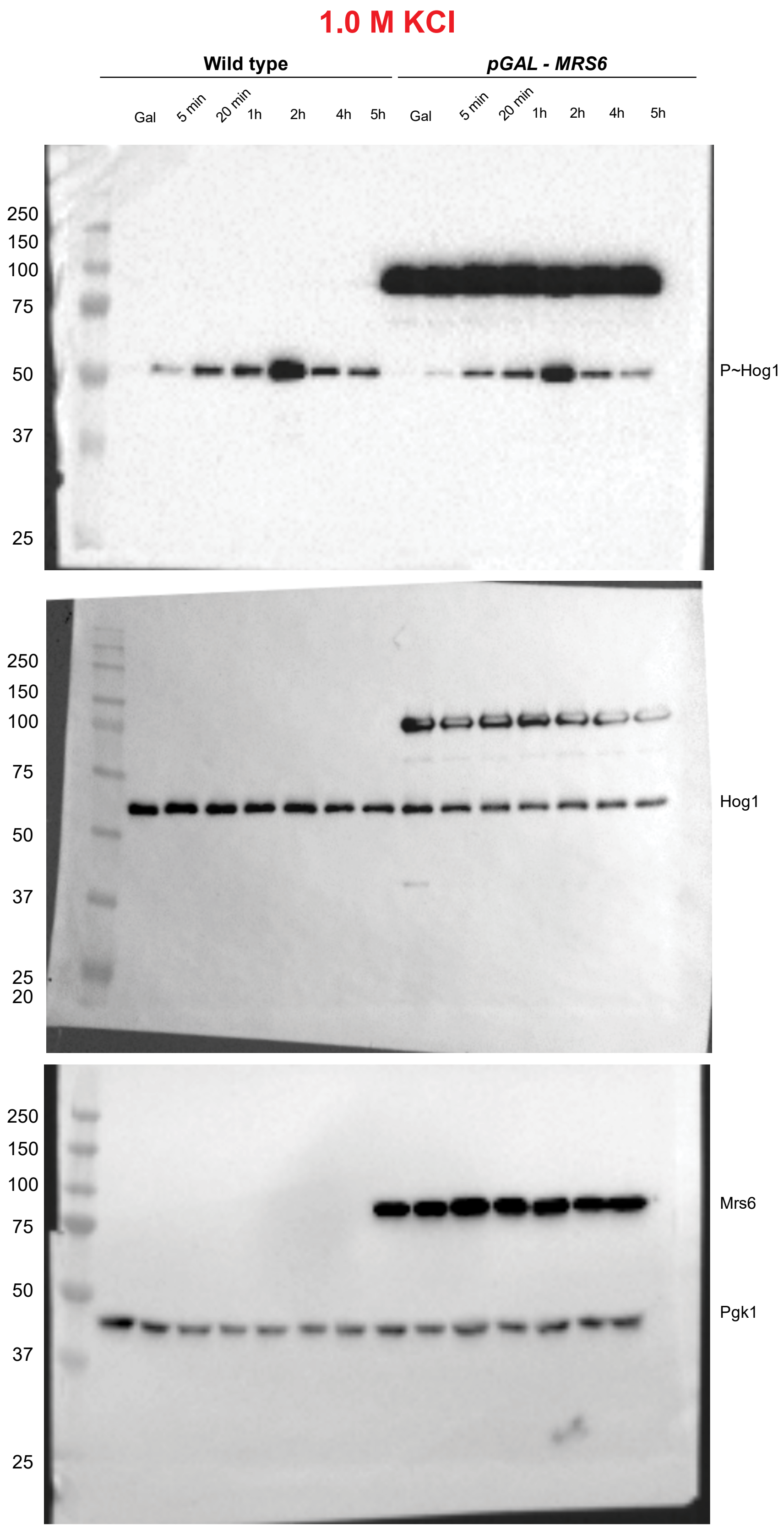

Figure S3C. Raw material for Fig. 6E.

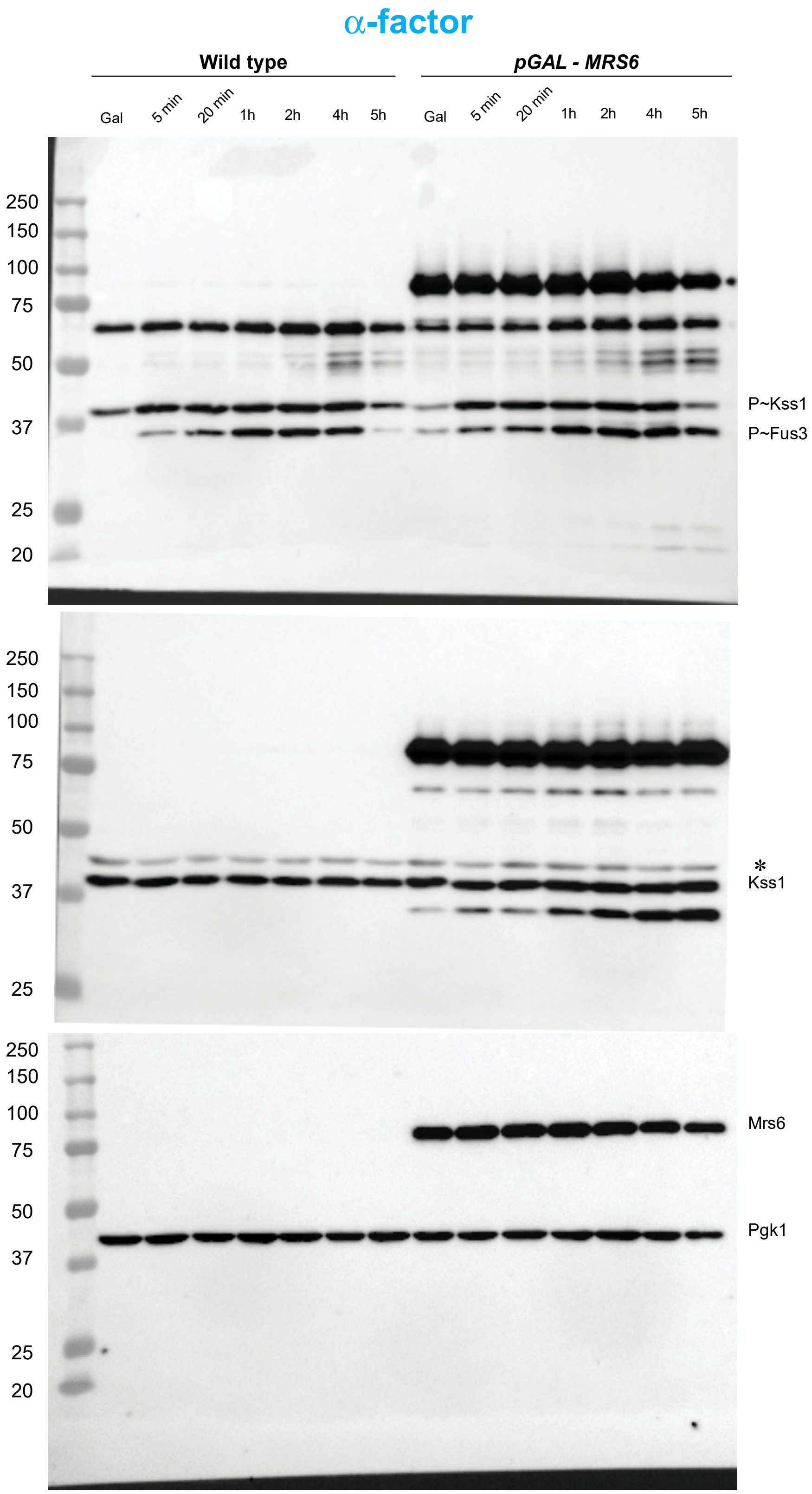

Table S1. Yeast strains used in the study.

Strain[a]	Genotype	Reference
PJ694A[b]	MAT**a** trpl-901 leu2-3,112 ura3-52 his3-200 gal4Δ gal80Δ LYS2::GALl-HIS3 GAL2-ADE2 met2::GAL7-lacZ	(James et al., 1996)
PC313	MAT**a** ura3-52	(Liu et al., 1993)
PC538	MAT**a** ste4 FUS1-lacZ FUS1-HIS3 ura3-52	(Cullen et al., 2004)
PC549	MAT**a** SY3089 ste4 FUS1-lacZ FUS1-HIS3 ura3-52 ste20::URA3	(Cullen and Sprague, 2000)
PC610	MAT**a** SY3089 ste4 FUS1-lacZ FUS1-HIS3 ura3-52 ste50::URA3	(Pitoniak et al., 2015)
PC999	MAT**a** ste4 FUS1-lacZ FUS1-HIS3 ura3-52 MSB2HA@500 aa	(Cullen et al., 2004)
PC3209	MAT**a** ste4 FUS1-lacZ FUS1-HIS3 ura3-52 msb2::KanMX6	(Cullen et al., 2004)
PC3551	MAT**a** ste4 FUS1-lacZ FUS1-HIS3 ura3-52 leu2::HYG bem4::NAT	(Pitoniak et al., 2015)
PC3861	MAT**a** ste11::NAT ste4 FUS1-lacZ FUS1-HIS3 ura3-52	(Karunanithi et al., 2010)
PC5024	MAT**a** ste11::NAT ura3-52	(Pitoniak et al., 2015)
PC5392	MAT**a** ste4 FUS1-lacZ FUS1-HIS3 ura3-52 STE7-GFP::KanMX6	This study
PC5692	MAT**a** ste4 FUS1-lacZ FUS1-HIS3 ura3-52 sho1::HYG	(Vadaie et al., 2008)
PC6604	MAT**a** ura3-52 leu2 ssk1 ste11::NAT	(Basu et al., 2020)
PC6785	MAT**a** ura3-52 leu2 ssk1 ste20::URA3	(Basu et al., 2020)
PC6810	MAT**a** ura3-52 leu2 ssk1 ura3-	(Basu et al., 2020)
PC3752	MAT**a** ste4 FUS1-lacZ FUS1-HIS3 ura3-52 opy2::URA3	(Karunanithi and Cullen, 2012)
PC7386	MAT**a** trpl-901 leu2-3,112 ura3-52 his3-200 gal4Δ gal80Δ LYS2::GALl-HIS3 GAL2-ADE2 met2::GAL7-lacZ pGAD-C1 and pGBDU-C1 [c]	This study
PC7387	MAT**a** trpl-901 leu2-3,112 ura3-52 his3-200 gal4Δ gal80Δ LYS2::GALl-HIS3 GAL2-ADE2 met2::GAL7-lacZ pGAD-C1 and pGBDU-C1-MRS6	This study
PC7388	MAT**a** trpl-901 leu2-3,112 ura3-52 his3-200 gal4Δ gal80Δ LYS2::GALl-HIS3 GAL2-ADE2 met2::GAL7-lacZ pGAD-C1 and pGBDU-C1-BEM4	This study
PC7389	MAT**a** trpl-901 leu2-3,112 ura3-52 his3-200 gal4Δ gal80Δ LYS2::GALl-HIS3 GAL2-ADE2 met2::GAL7-lacZ pGAD-C1-CDC42 and pGBDU-C1	This study
PC7390	MAT**a** trpl-901 leu2-3,112 ura3-52 his3-200 gal4Δ gal80Δ LYS2::GALl-HIS3 GAL2-ADE2 met2::GAL7-lacZ pGAD-C1-CDC42 and pGBDU-C1-MRS6	This study
PC7391	MAT**a** trpl-901 leu2-3,112 ura3-52 his3-200 gal4Δ gal80Δ LYS2::GALl-HIS3 GAL2-ADE2 met2::GAL7-lacZ pGAD-C1-CDC42 and pGBDU-C1-BEM4	This study
PC7393	MAT**a** trpl-901 leu2-3,112 ura3-52 his3-200 gal4Δ gal80Δ LYS2::GALl-HIS3 GAL2-ADE2 met2::GAL7-lacZ pGBDU-C1-CDC42 and pGAD-C1	This study
PC7394	MAT**a** trpl-901 leu2-3,112 ura3-52 his3-200 gal4Δ gal80Δ LYS2::GALl-HIS3 GAL2-ADE2 met2::GAL7-lacZ pGBDU-C1-CDC42 and pGAD-C1-MRS6	This study
PC7395	MAT**a** trpl-901 leu2-3,112 ura3-52 his3-200 gal4Δ gal80Δ LYS2::GALl-HIS3 GAL2-ADE2 met2::GAL7-lacZ pGAD-C1-MRS6 and pGBDU-C1-STE11	This study
PC7396	MAT**a** trpl-901 leu2-3,112 ura3-52 his3-200 gal4Δ gal80Δ LYS2::GALl-HIS3 GAL2-ADE2 met2::GAL7-lacZ pGAD-C1 and pGBDU-C1-STE11	This study
PC7397	MAT**a** trpl-901 leu2-3,112 ura3-52 his3-200 gal4Δ gal80Δ LYS2::GALl-HIS3 GAL2-ADE2 met2::GAL7-lacZ pGAD-C1-MRS6 and pGBDU-C1-STE20	This study
PC7398	MAT**a** trpl-901 leu2-3,112 ura3-52 his3-200 gal4Δ gal80Δ LYS2::GALl-HIS3 GAL2-ADE2 met2::GAL7-lacZ pGAD-C1 and pGBDU-C1-STE20	This study
PC7399	MAT**a** trpl-901 leu2-3,112 ura3-52 his3-200 gal4Δ gal80Δ LYS2::GALl-HIS3 GAL2-ADE2 met2::GAL7-lacZ pGAD-C1-MRS6 and pGBDU-C1-STE50	This study
PC7400	MAT**a** trpl-901 leu2-3,112 ura3-52 his3-200 gal4Δ gal80Δ LYS2::GALl-HIS3 GAL2-ADE2 met2::GAL7-lacZ pGAD-C1 and pGBDU-C1-STE50	This study
PC7401	MAT**a** trpl-901 leu2-3,112 ura3-52 his3-200 gal4Δ gal80Δ LYS2::GALl-HIS3 GAL2-ADE2 met2::GAL7-lacZ pGAD-C1-MRS6 and pGBDU-C1-RDI1	This study
PC7402	MAT**a** trpl-901 leu2-3,112 ura3-52 his3-200 gal4Δ gal80Δ LYS2::GALl-HIS3 GAL2-ADE2 met2::GAL7-lacZ pGAD-C1 and pGBDU-C1-RDI1	This study
PC7403	MAT**a** trpl-901 leu2-3,112 ura3-52 his3-200 gal4Δ gal80Δ LYS2::GALl-HIS3 GAL2-ADE2 met2::GAL7-lacZ pGAD-C1-MRS6 and pGBDU-C1-STE4	This study
PC7404	MAT**a** trpl-901 leu2-3,112 ura3-52 his3-200 gal4Δ gal80Δ LYS2::GALl-HIS3 GAL2-ADE2 met2::GAL7-lacZ pGAD-C1 and pGBDU-C1-STE4	This study
PC7405	MAT**a** trpl-901 leu2-3,112 ura3-52 his3-200 gal4Δ gal80Δ LYS2::GALl-HIS3 GAL2-ADE2 met2::GAL7-lacZ pGAD-C1-MRS6 and pGBDU-C1-STE7	This study
PC7406	MAT**a** trpl-901 leu2-3,112 ura3-52 his3-200 gal4Δ gal80Δ LYS2::GALl-HIS3 GAL2-ADE2 met2::GAL7-lacZ pGAD-C1 and pGBDU-C1-STE7	This study
PC7407	MAT**a** trpl-901 leu2-3,112 ura3-52 his3-200 gal4Δ gal80Δ LYS2::GALl-HIS3 GAL2-ADE2 met2::GAL7-lacZ pGAD-C1-MRS6 and pGBDU-C1-STE11	This study
PC7408	MAT**a** trpl-901 leu2-3,112 ura3-52 his3-200 gal4Δ gal80Δ LYS2::GALl-HIS3 GAL2-ADE2 met2::GAL7-lacZ pGAD-C1 and pGBDU-C1-STE11	This study
PC7409	MAT**a** trpl-901 leu2-3,112 ura3-52 his3-200 gal4Δ gal80Δ LYS2::GALl-HIS3 GAL2-ADE2 met2::GAL7-lacZ pGAD-C1-MRS6 and pGBDU-C1-STE20	This study
PC7410	MAT**a** trpl-901 leu2-3,112 ura3-52 his3-200 gal4Δ gal80Δ LYS2::GALl-HIS3 GAL2-ADE2 met2::GAL7-lacZ pGAD-C1 and pGBDU-C1-STE20	This study
PC7413	MAT**a** trpl-901 leu2-3,112 ura3-52 his3-200 gal4Δ gal80Δ LYS2::GALl-HIS3 GAL2-ADE2 met2::GAL7-lacZ pGAD-C1-MRS6 and pGBDU-C1-CDC24	This study
PC7414	MAT**a** trpl-901 leu2-3,112 ura3-52 his3-200 gal4Δ gal80Δ LYS2::GALl-HIS3 GAL2-ADE2 met2::GAL7-lacZ pGAD-C1 and pGBDU-C1-CDC24	This study
PC7417	MAT**a** trpl-901 leu2-3,112 ura3-52 his3-200 gal4Δ gal80Δ LYS2::GALl-HIS3 GAL2-ADE2 met2::GAL7-lacZ pGAD-C1-MRS6 and pGBDU-C1-OPY2	This study
PC7418	MAT**a** trpl-901 leu2-3,112 ura3-52 his3-200 gal4Δ gal80Δ LYS2::GALl-HIS3 GAL2-ADE2 met2::GAL7-lacZ pGAD-C1 and pGBDU-C1-OPY2	This study
PC7419	MAT**a** trpl-901 leu2-3,112 ura3-52 his3-200 gal4Δ gal80Δ LYS2::GALl-HIS3 GAL2-ADE2 met2::GAL7-lacZ pGAD-C1-MRS6 and pGBDU-C1-SSK1	This study
PC7420	MAT**a** trpl-901 leu2-3,112 ura3-52 his3-200 gal4Δ gal80Δ LYS2::GALl-HIS3 GAL2-ADE2 met2::GAL7-lacZ pGAD-C1 and pGBDU-C1-SSK1	This study
PC7421	MAT**a** trpl-901 leu2-3,112 ura3-52 his3-200 gal4Δ gal80Δ LYS2::GALl-HIS3 GAL2-ADE2 met2::GAL7-lacZ pGAD-C1-MRS6 and pGBDU-C1-KSS1	This study
PC7422	MAT**a** trpl-901 leu2-3,112 ura3-52 his3-200 gal4Δ gal80Δ LYS2::GALl-HIS3 GAL2-ADE2 met2::GAL7-lacZ pGAD-C1 and pGBDU-C1-KSS1	This study
PC7425	MAT**a** trpl-901 leu2-3,112 ura3-52 his3-200 gal4Δ gal80Δ LYS2::GALl-HIS3 GAL2-ADE2 met2::GAL7-lacZ pGAD-C1-MRS6 and pGBDU-C1-BEM1	This study
PC7426	MAT**a** trpl-901 leu2-3,112 ura3-52 his3-200 gal4Δ gal80Δ LYS2::GALl-HIS3 GAL2-ADE2 met2::GAL7-lacZ pGAD-C1 and pGBDU-C1-BEM1	This study
PC7427	MAT**a** trpl-901 leu2-3,112 ura3-52 his3-200 gal4Δ gal80Δ LYS2::GALl-HIS3 GAL2-ADE2 met2::GAL7-lacZ pGAD-C1-MRS6 and pGBDU-C1-BEM4	This study
PC7428	MAT**a** trpl-901 leu2-3,112 ura3-52 his3-200 gal4Δ gal80Δ LYS2::GALl-HIS3 GAL2-ADE2 met2::GAL7-lacZ pGAD-C1 and pGBDU-C1-BEM4	This study
PC7429	MAT**a** trpl-901 leu2-3,112 ura3-52 his3-200 gal4Δ gal80Δ LYS2::GALl-HIS3 GAL2-ADE2 met2::GAL7-lacZ pGAD-C1-MRS6 and pGBDU-C1-IRE1	This study
PC7430	MAT**a** trpl-901 leu2-3,112 ura3-52 his3-200 gal4Δ gal80Δ LYS2::GALl-HIS3 GAL2-ADE2 met2::GAL7-lacZ pGAD-C1 and pGBDU-C1-IRE1	This study
PC7431	MAT**a** trpl-901 leu2-3,112 ura3-52 his3-200 gal4Δ gal80Δ LYS2::GALl-HIS3 GAL2-ADE2 met2::GAL7-lacZ pGAD-C1-MRS6	This study
PC7439	MAT**a** ste4 FUS1-lacZ FUS1-HIS3 ura3-52 pRS316 [d]	This study
PC7440	MAT**a** ste4 FUS1-lacZ FUS1-HIS3 ura3-52 pGAL-BMH1[e]	This study
PC7441	MAT**a** ste4 FUS1-lacZ FUS1-HIS3 ura3-52 pGAL-BMH2[e]	This study
PC7442	MAT**a** ste4 FUS1-lacZ FUS1-HIS3 ura3-52 pGAL-MRS6 [e]	This study
PC7443	MAT**a** ste4 FUS1-lacZ FUS1-HIS3 ura3-52 pGAL-KAR2 [e]	This study
PC7444	MAT**a** ura3-52 leu2 ssk1 pRS316 [d]	This study
PC7445	MAT**a** ura3-52 leu2 ssk1 pGAL-BMH1 [e]	This study
PC7446	MAT**a** ura3-52 leu2 ssk1 pGAL-BMH2 [e]	This study
PC7447	MAT**a** ura3-52 leu2 ssk1 pGAL-MRS6 [e]	This study
PC7448	MAT**a** ura3-52 leu2 ssk1 pGAL-KAR2 [e]	This study
PC7589	MAT**a** ura3-52 CDC3-mCherry::HYG + GFP-CDC42 + pRS316	This study
PC7590	MAT**a** ura3-52 CDC3-mCherry::HYG + GFP-CDC42 + pGAL-MRS6	This study
PC7592	MAT**a** ura3-52 CDC3-mCherry::HYG tec1::NAT + GFP-Cdc42 + pGAL-MRS6	This study

[a] Strains are in the ∑1287b background unless otherwise indicated.

[b] This strain comes from the laboratory of M. Johnston (University of Colorado Medical Center in Denver).

[c] pGAD-C1 and PGBDU-C1 and plasmids have been described (James et al. 1996).

[d] pRS vectors are from (Sikorski and Hieter1989).

[e] *pGAL* plasmids are from (Gelperin et al. 2005).

Table S2 [a]. Analysis of the genome-wide overexpression screen.

Plate[a]	Row	Column	Gene ID	Gene Name [b]	Intensity[c]
20	A	5	*YOR202W*	*HIS3*	245
38	D	1	*YJL077C*	*ICS3*	226
42	B	2	*YHR213W-A*	*YHR213W-A*	186
30	F	7	*YOR370C*	*MRS6*	174
23	A	5	*YER177W*	*BMH1*	165
52	B	4	*YGR044C*	*RME1*	158
28	B	5	*YML116W*	*ATR1*	155
52	A	1	*YOR251C*	*TUM1*	154
52	D	1	*YPR151C*	*SUE1*	153
52	B	2	*YDL095W*	*PMT1*	151
52	A	5	*YDR418W*	*RPL12B*	150
28	C	10	*YOR028C*	*CIN5*	147
52	C	1	*YHR215W*	*PHO12*	139
52	B	3	*YDR529C*	*QCR7*	137
52	B	1	*YAR071W*	*PHO11*	136
32	C	5	*YOR212W*	*STE4*	134
52	A	3	*YLL010C*	*PSR1*	134
52	A	7	*YPL080C*	*YPL080C*	131
36	A	9	*YDR099W*	*BMH2*	131
41	D	3	*YDR152W*	*GIR2*	128
19	B	4	*YGL121C*	*GPG1*	122
42	F	2	*YIL028W*	*RPL13A*	118
52	D	5	*YGL030W*	*YIL028W*	116
56	E	8	*YDL082W*	*RPL30*	115
42	A	1	*YHL002W*	*HSE1*	115
52	C	2	*YLR209C*	*PNP1*	115
51	G	4	*YDL159W*	*STE7*	110
27	H	10	*YOR336W*	*KRE5*	109
52	A	6	*YDL191W*	*RPL35A*	106
38	E	4	*YOL085C*	*YOL085C*	104
21	C	10	*YGL209W*	*MIG2*	102
24	A	6	*YAL027W*	*SAW1*	102
37	B	1	*YBR021W*	*FUR4*	101
27	B	11	*YDR303C*	*RSC3*	100
32	B	7	*YKL028W*	*TFA1*	97
42	A	3	*YIL060W*	*YIL060W*	96
-	-	-	-	*N.D.*	94
-	-	-	-	*N.D.*	92
37	E	2	*YBL014C*	*RRN6*	85
52	F	3	*YDL138W*	*RGT2*	81
38	A	3	*YJL034W*	*KAR2*	80
4	F	1	*YDR510C-A*	*YDR510C-A*	73
44	E	11	*YDR519W*	*FPR2*	68
27	F	12	*YPR070W*	*MED1*	68
31	C	8	*YDR182W*	*CDC1*	67
4	D	3	*YHR125W*	*YHR125W*	67
61	A	7	*YOR216C*	*RUD3*	64
23	H	8	*YMR251W-A*	*HOR7*	62
46	B	1	*YML118W*	*NGL3*	62

Footnotes

[a]The data file comes from (Gelperin et al., 2005). Blank lines in the data file indicate wells for which the clone identity could not be determined after two rounds of sequencing.

[b]Gene names for the top 200 candidates are shown.

[c]Spot intensity was measured by ImageJ analysis.

Table S3. Validation of candidates from the screen by plasmid transformation into wild-type cells and the *msb2*Δ mutant.

Screen Validation in WT[a]		Normalized Growth Intensity in WT [c]	Growth Intensity in WT Normalized to *msb2*Δ [d]
**Gene**	**Intensity[b]**		
HIS3	204	4	22
STE4	184	3	20
STE7	167	3	18
BMH1	165	3	18
BMH2	151	3	16
MIG2	147	3	16
RRN6	146	3	16
MRS6	137	2	15
CIN5	132	2	14
KAR2	130	2	14
TFA1	123	2	13
RSC3	120	2	13
RGT2	113	2	12
ATR1	104	2	11
WT	57	1	6
RPL12B	57	1	6
FUR4	49	1	5
YHR125W	47	1	5
WT	30	1	3
RPL13A	24	<0.1	3
GPG1	21	<0.1	2
YIL060W	15	<0.1	2
ICS3	11	<0.1	1
HSE1	9	<0.1	1
YPL080C	8	<0.1	1
RPL21A	2	<0.1	<0.1
RPL30	2	<0.1	<0.1
FPR2	2	<0.1	<0.1
QCR7	2	<0.1	<0.1
RME1	2	<0.1	<0.1
MED1	1	<0.1	<0.1
YOL085C	1	<0.1	<0.1
YHR213W-A	1	<0.1	<0.1
GIR2	1	<0.1	<0.1
RPL35A	1	<0.1	<0.1
PMT1	1	<0.1	<0.1
YIL028W	<0.1	<0.1	<0.1
PHO11	<0.1	<0.1	<0.1
SUE1	<0.1	<0.1	<0.1
QRI1	<0.1	<0.1	<0.1
KRE5	<0.1	<0.1	<0.1
PNP1	<0.1	<0.1	<0.1
TUM1	<0.1	<0.1	<0.1
PHO12	<0.1	<0.1	<0.1
CDC1	<0.1	<0.1	<0.1
PSR1	<0.1	<0.1	<0.1

Screen Validation in *msb2*Δ[e]		Normalized Growth Intensity in *msb2*Δ [c]
**Gene**	**Intensity[f]**	
HIS3	199	22
STE4	181	20
STE7	117	13
BMH1	77	9
BMH2	16	2
MIG2	56	6
RRN6	22	2
MRS6	14	2
CIN5	16	2
KAR2	33	4
TFA1	<0.1	<0.1
RSC3	<0.1	<0.1
RGT2	<0.1	<0.1
ATR1	<0.1	<0.1
WT	9	1
RPL12B	<0.1	<0.1
FUR4	<0.1	<0.1
YHR125W	<0.1	<0.1
WT	<0.1	<0.1
RPL13A	<0.1	<0.1
GPG1	<0.1	<0.1
YIL060W	<0.1	<0.1
ICS3	<0.1	<0.1
HSE1	<0.1	<0.1
YPL080C	<0.1	<0.1
RPL21A	<0.1	<0.1
RPL30	<0.1	<0.1
FPR2	<0.1	<0.1
QCR7	<0.1	<0.1
RME1	<0.1	<0.1
MED1	<0.1	<0.1
YOL085C	<0.1	<0.1
YHR213W-A	17	2
GIR2	<0.1	<0.1
RPL35A	27	3
PMT1	<0.1	<0.1
YIL028W	<0.1	<0.1
PHO11	<0.1	<0.1
SUE1	1	<0.1
QRI1	<0.1	<0.1
KRE5	3	<0.1
PNP1	<0.1	<0.1
TUM1	<0.1	<0.1
PHO12	<0.1	<0.1
CDC1	<0.1	<0.1
PSR1	<0.1	<0.1

Screen Validation in WT[a]

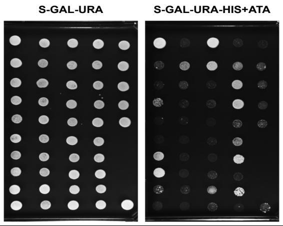

Screen Validation in *msb2*Δ[c]

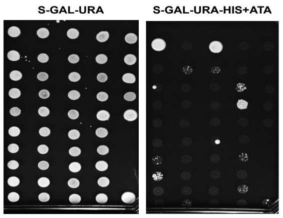

Validation Key

HIS3	*PMT1*	*STE4*	*YOL085C*	*QRI1*
ICS3	*CIN5*	*BMH2*	*RSC3*	*YHR125W*
YHR213W-A	*HSE1*	*YIL028W*	*MIG2*	*GPG1*
ATR1	*GIR2*	*RPL30*	*STE7*	*RPL21A*
RME1	*QCR7*	*PNP1*	*FUR4*	WT
TUM1	*PHO12*	*RPL35A*	*TFA1*	blank
MRS6	*PHO11*	*CDC1*	*RRN6*	blank
BMH1	*PSR1*	*YIL060W*	*MED1*	blank
RPL12B	*YPL080C*	*RGT2*	*KAR2*	blank
SUE1	*KRE5*	*RPL13A*	*FPR2*	WT

[a]The plasmids were transformed to PC6021 background.

[b]Colonies growth intensity measured in day 3.

[c]Values are normalized to WT's value. Wt is set to 1.

[d]Growth intensity rates of the canididates validated in wild type is normalized to *msb2*Δ mutant.

[e]The plasmids were transformed to PC3209 background.

[f]Colonies growth intensity measured in day 6.

Table S4. Raw data of genome-wide screen.

Left plate, S-GAL-URA; **middle** plate S-GAL-URA-HIS; and **right** plate S-GAL-URA-HIS+ATA.

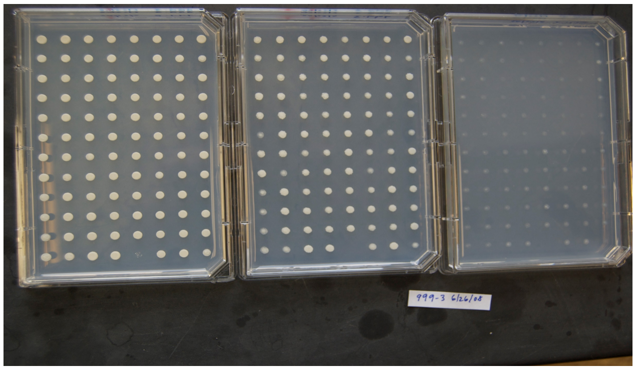

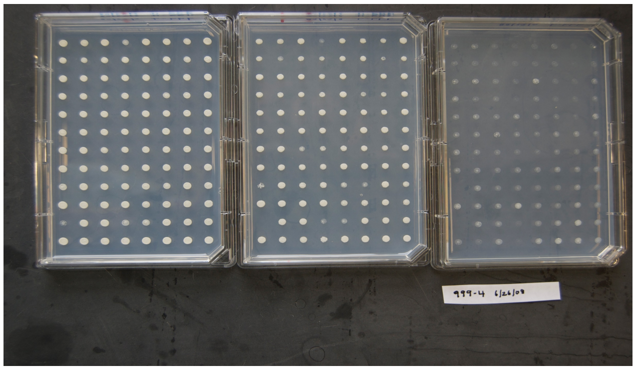

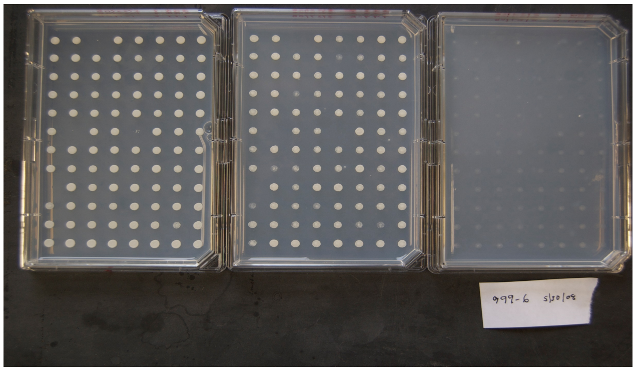

Left plate, S-GAL-URA; **middle** plate S-GAL-URA-HIS; and **right** plate S-GAL-URA-HIS+ATA.

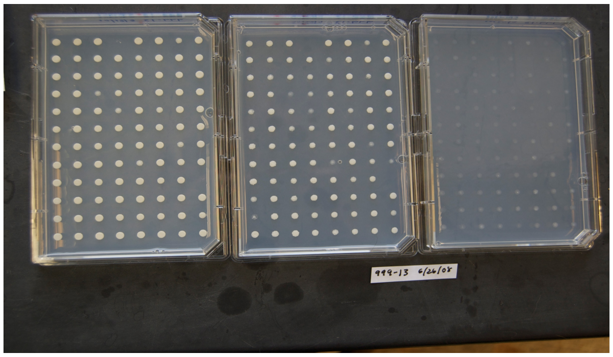

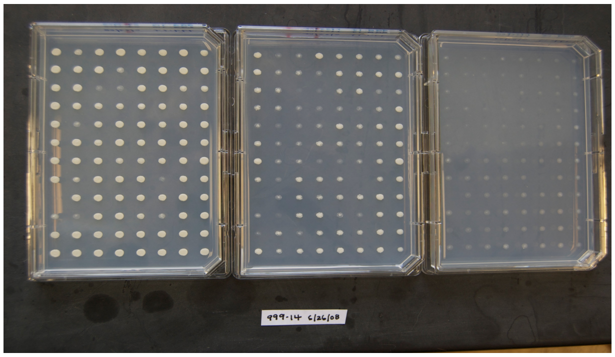

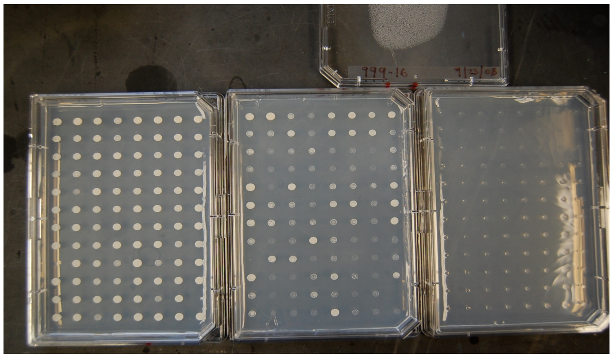

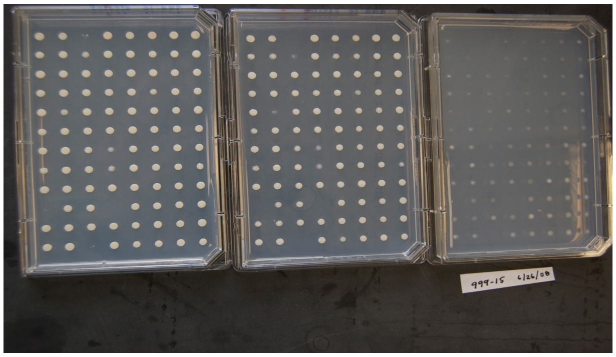

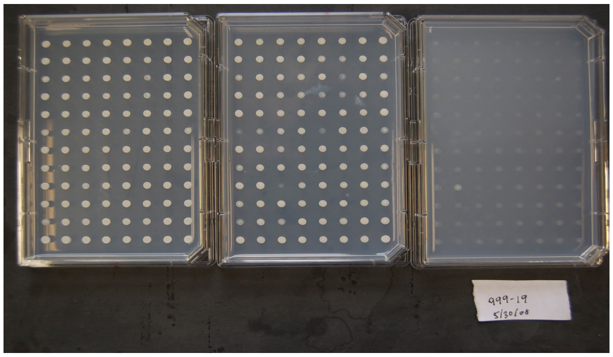

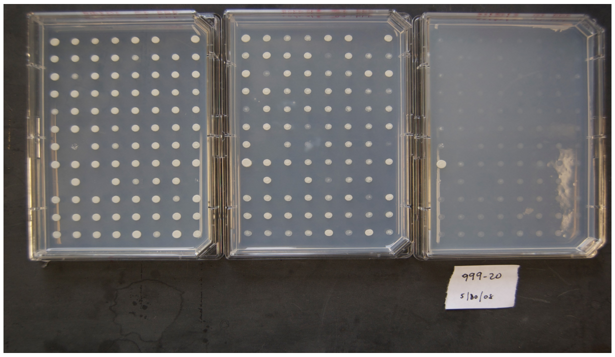

Left plate, S-GAL-URA; **middle** plate S-GAL-URA-HIS; and **right** plate S-GAL-URA-HIS+ATA.

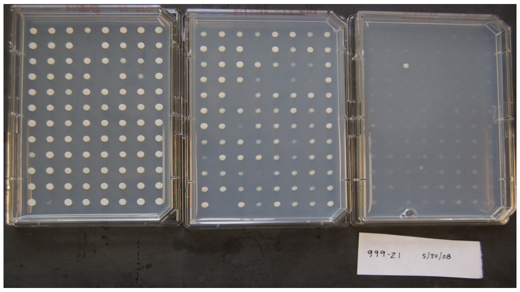

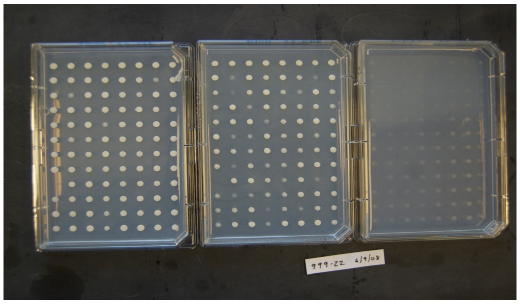

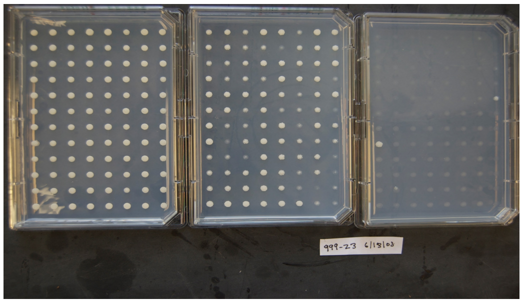

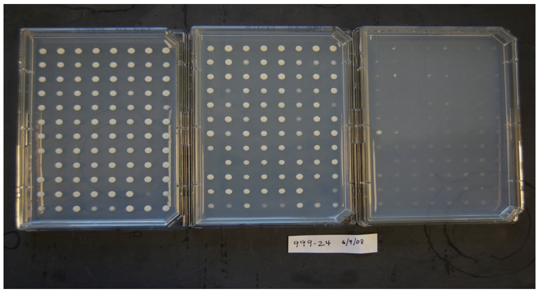

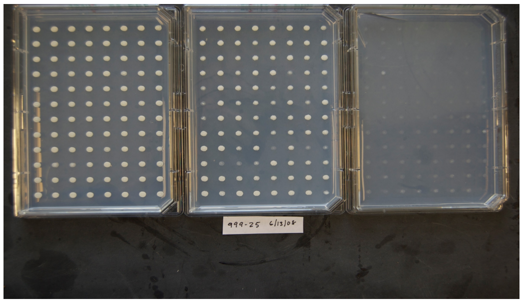

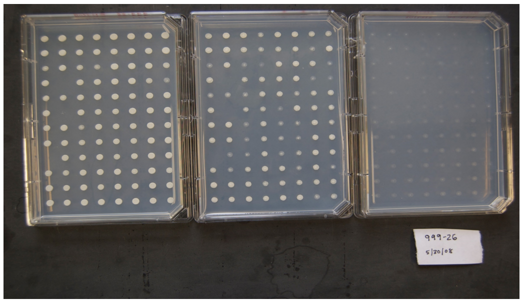

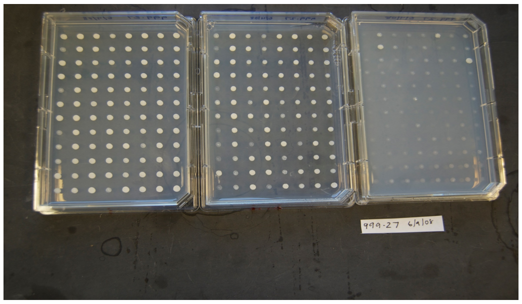

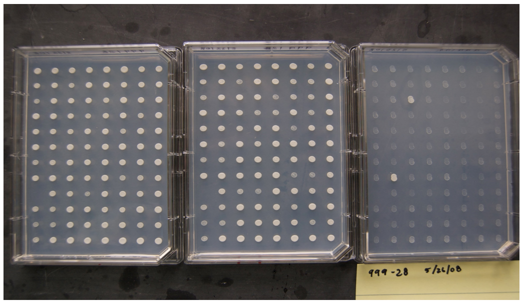

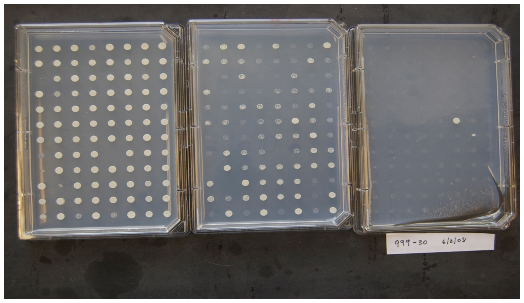

Left plate, S-GAL-URA; **middle** plate S-GAL-URA-HIS; and **right** plate S-GAL-URA-HIS+ATA.

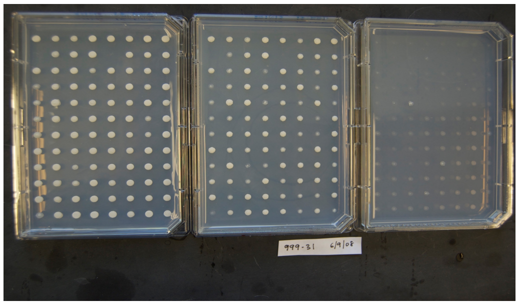

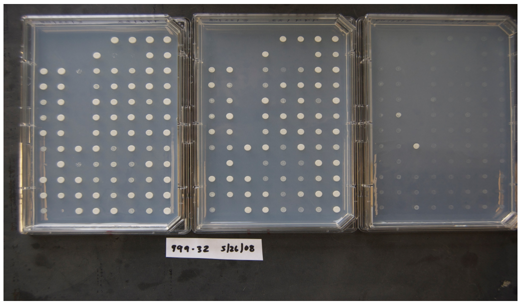

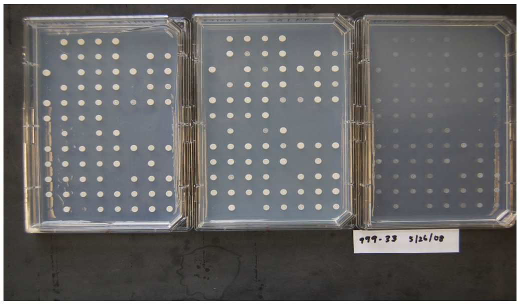

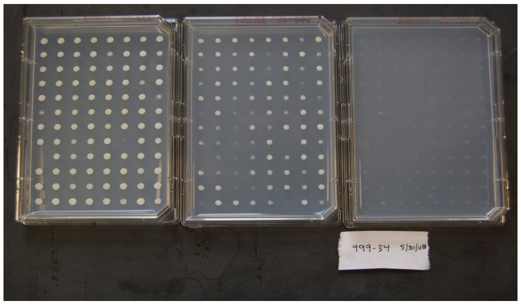

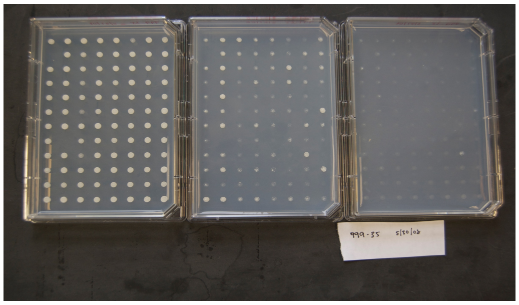

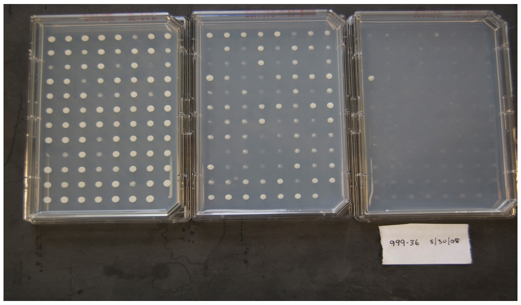

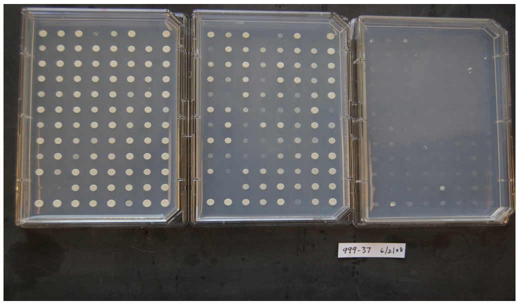

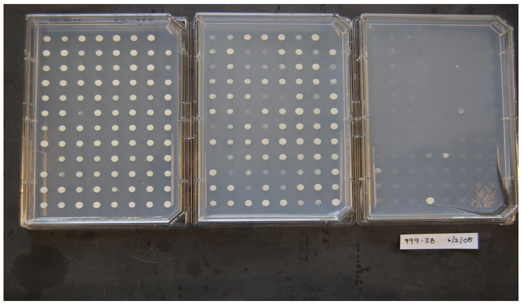

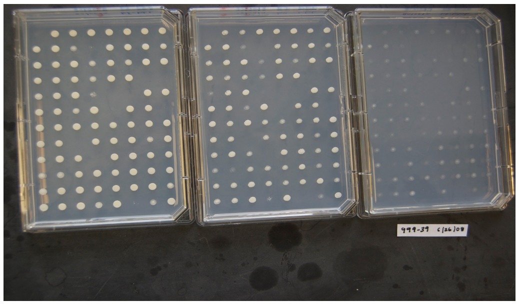

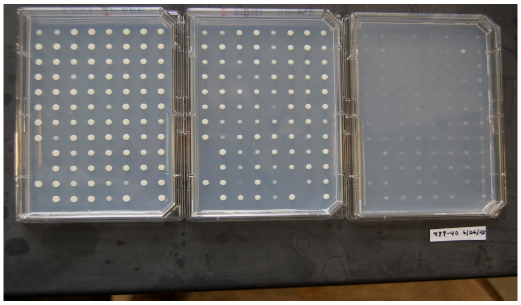

Left plate, S-GAL-URA; **middle** plate S-GAL-URA-HIS; and **right** plate S-GAL-URA-HIS+ATA.

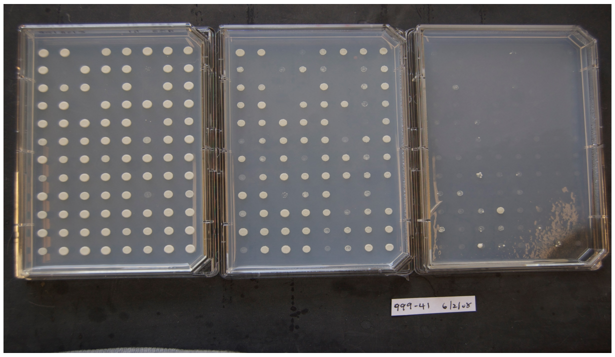

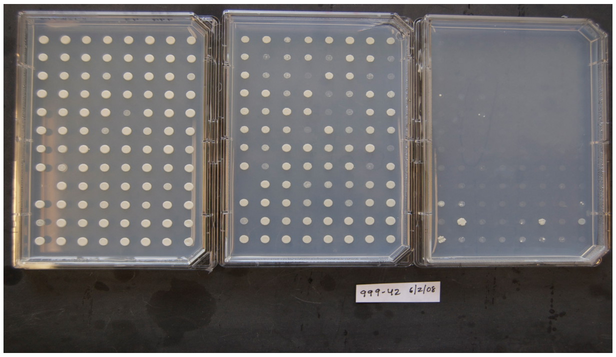

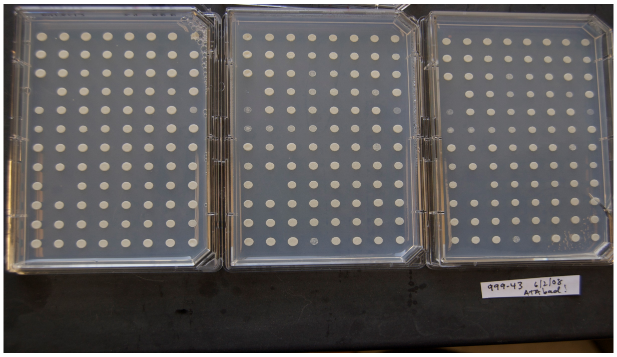

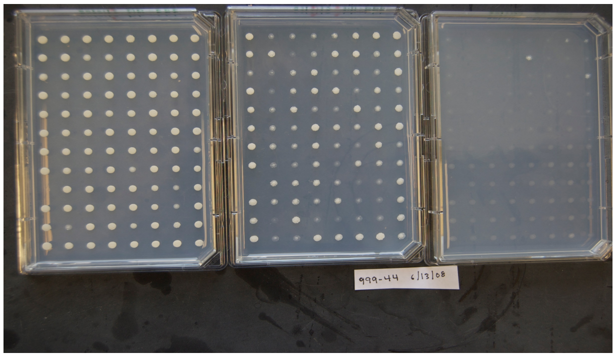

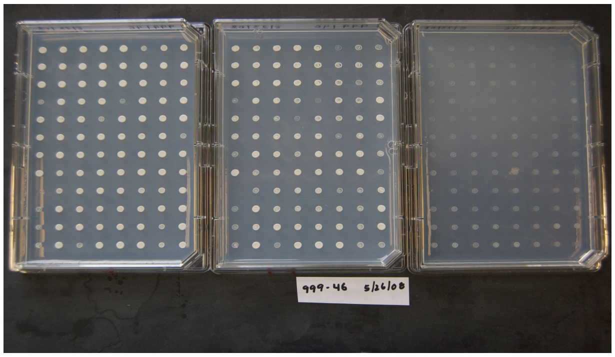

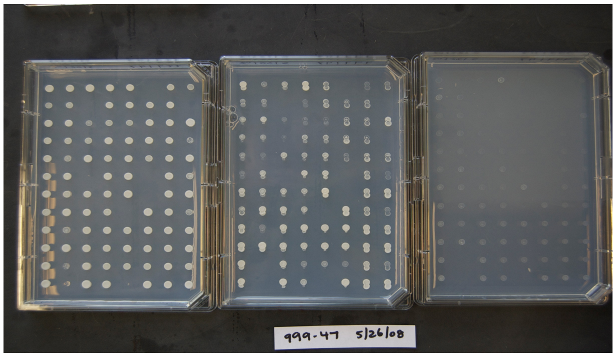

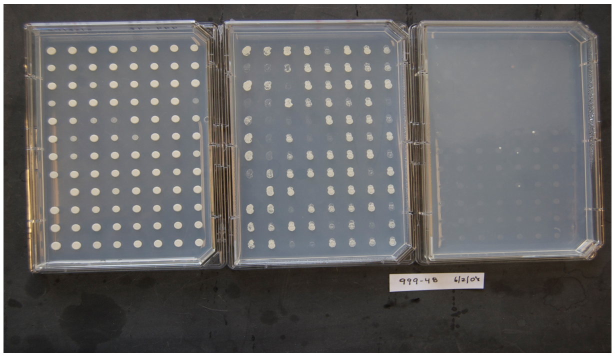

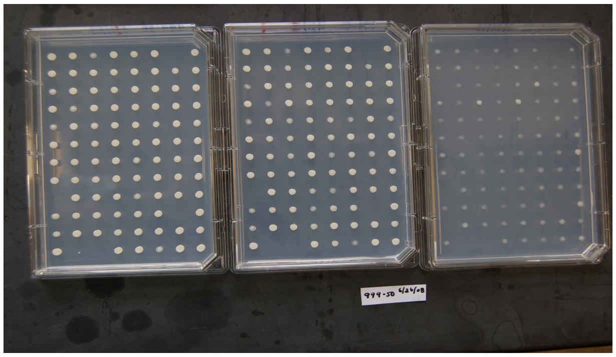

Left plate, S-GAL-URA; **middle** plate S-GAL-URA-HIS; and **right** plate S-GAL-URA-HIS+ATA.

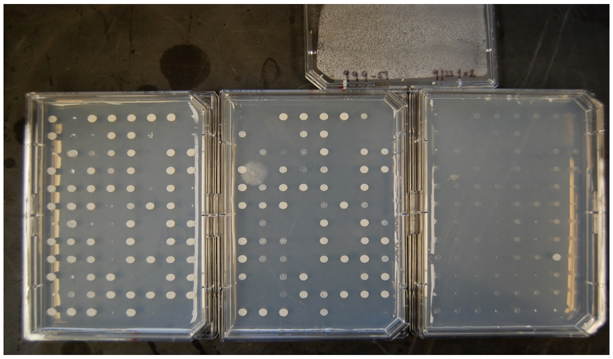

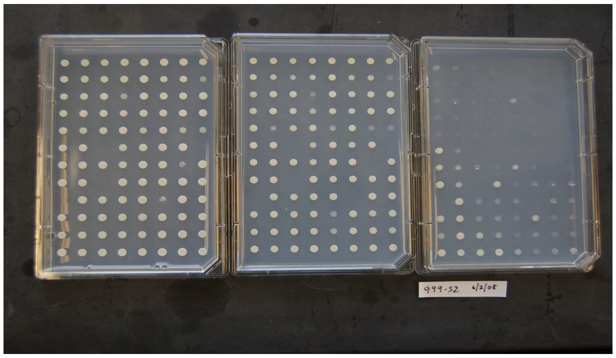

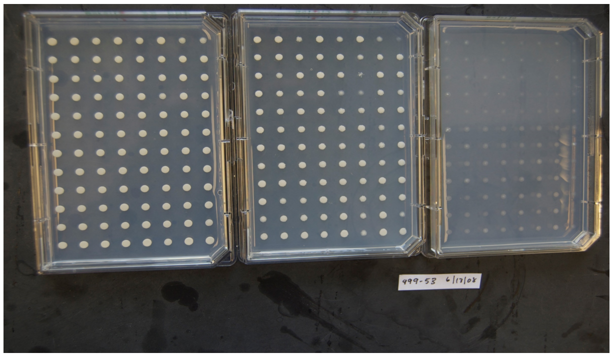

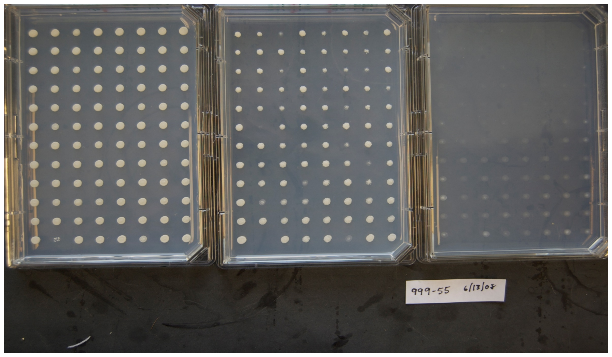

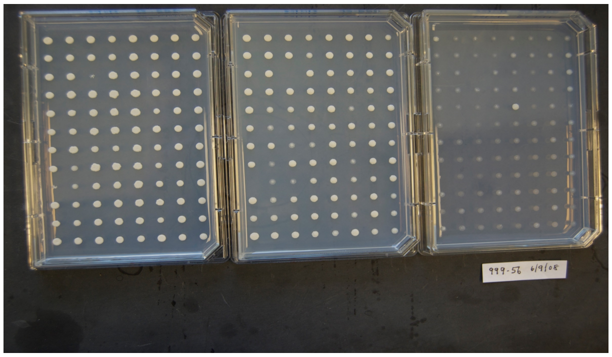

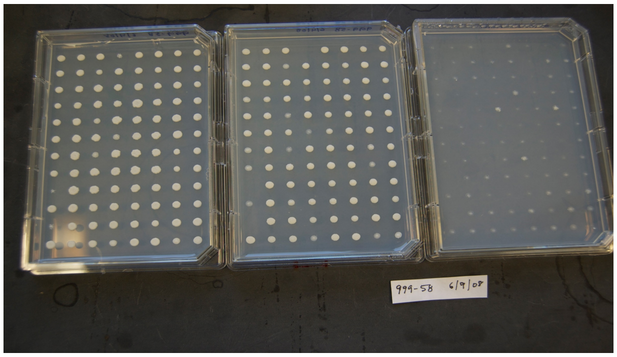

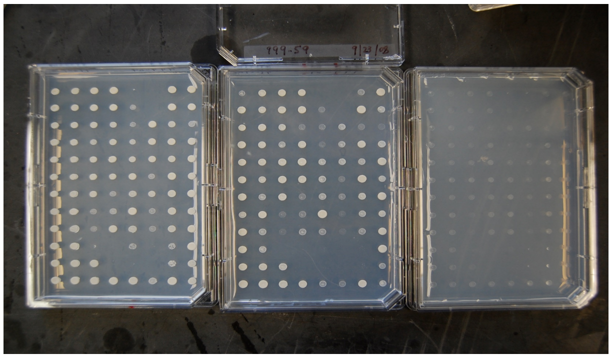

Left plate, S-GAL-URA; **middle** plate S-GAL-URA-HIS; and **right** plate S-GAL-URA-HIS+ATA.

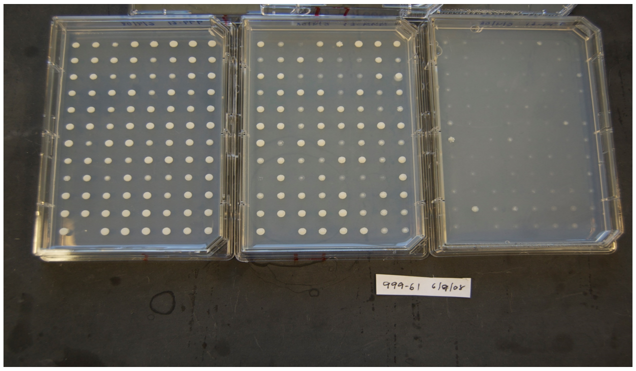

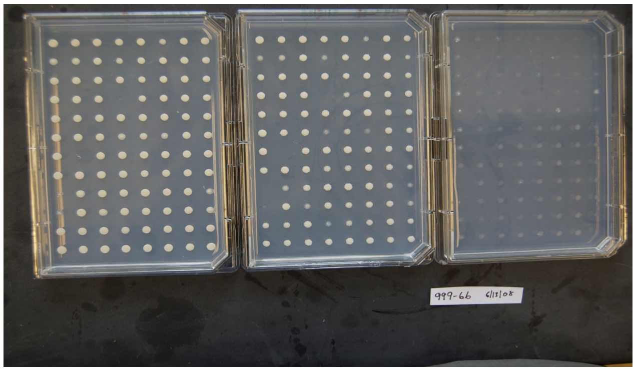

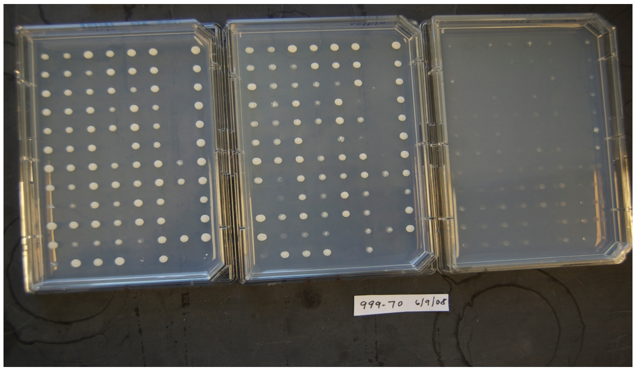

Supplement: Supplementary file 1 — Supplementary Information. [file 41598_2020_78470_MOESM1_ESM.pdf]
