# Supplementary figures and images for: A Rab escort protein regulates the MAPK pathway that controls filamentous growth in yeast
Source: Sci Rep. 2020 Dec 17;10:22184. doi: 10.1038/s41598-020-78470-4 (PMC7746766; doi:10.1038/s41598-020-78470-4)

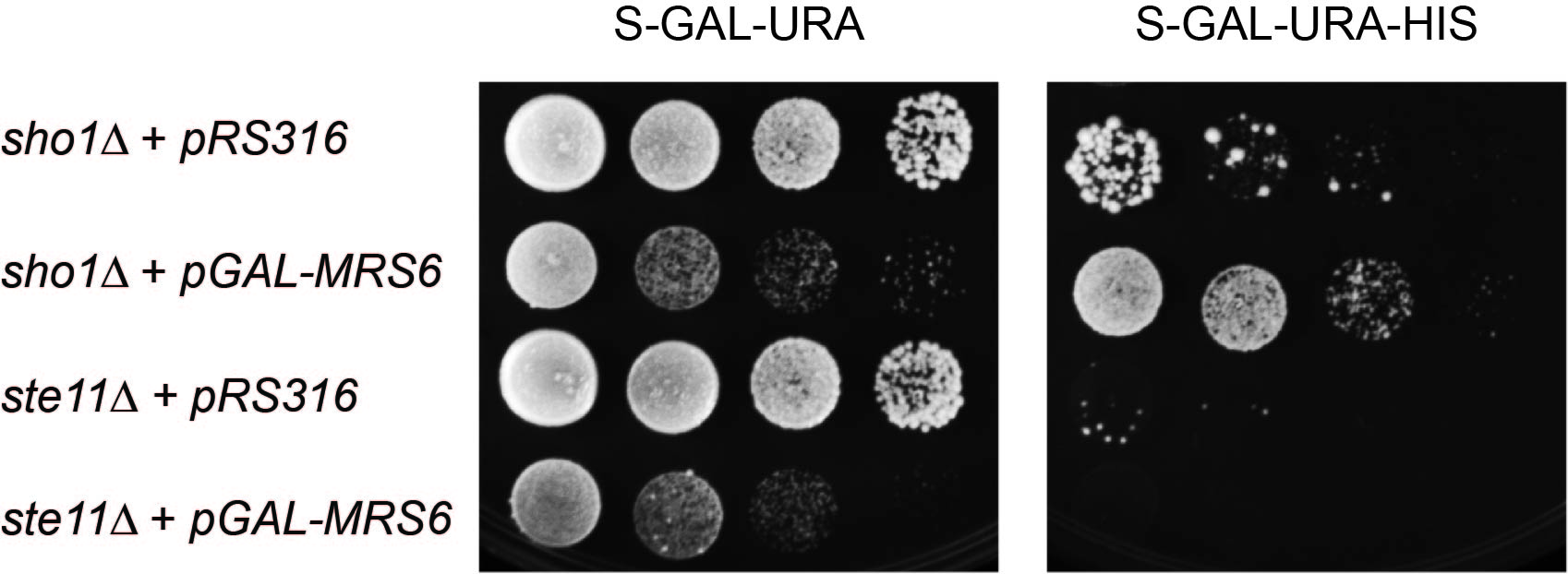

Supplement: Supplementary file 6 — Supplementary Figure s1. [file 41598_2020_78470_MOESM6_ESM.jpg]

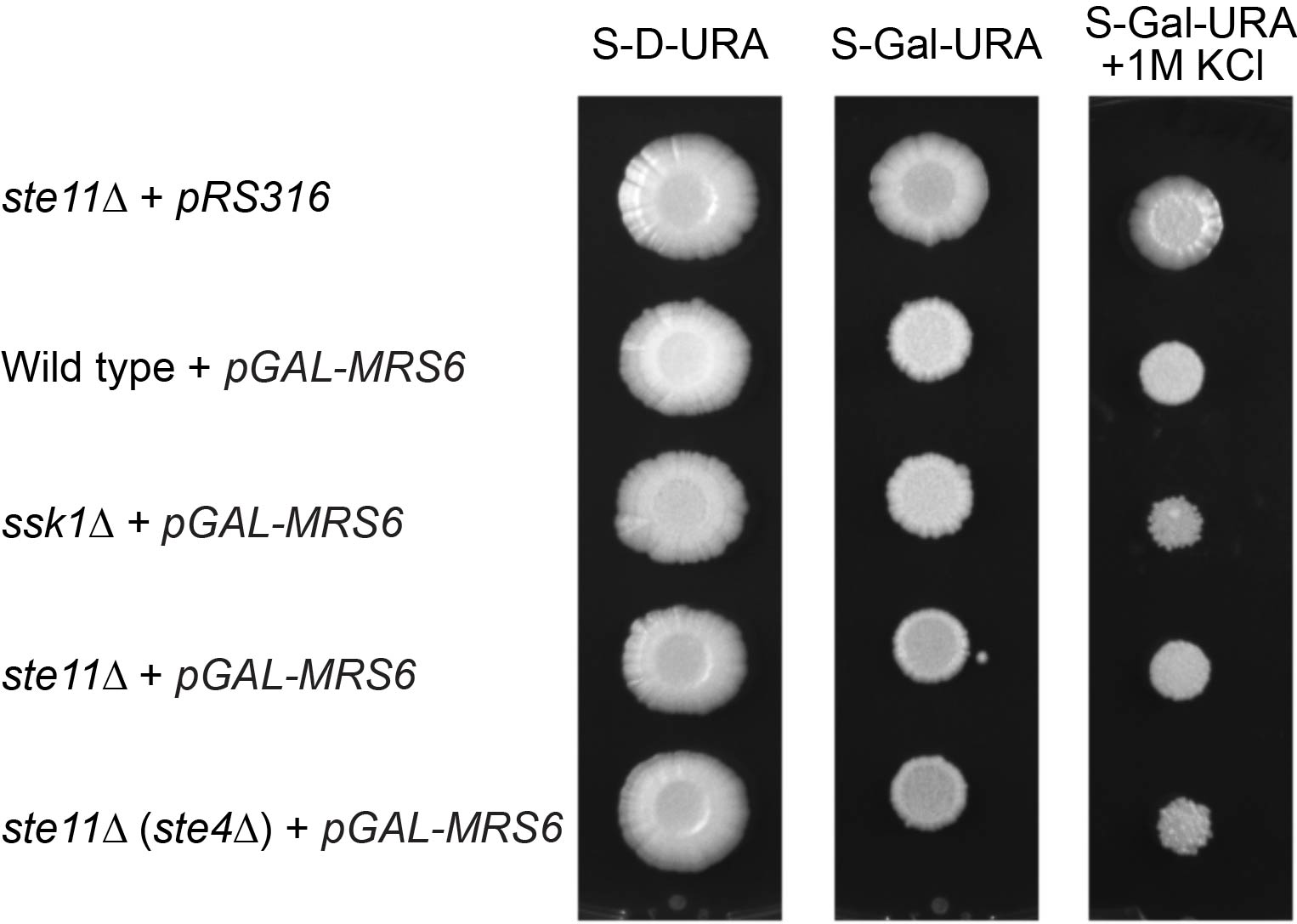

Supplement: Supplementary file 7 — Supplementary Figure s2. [file 41598_2020_78470_MOESM7_ESM.jpg]

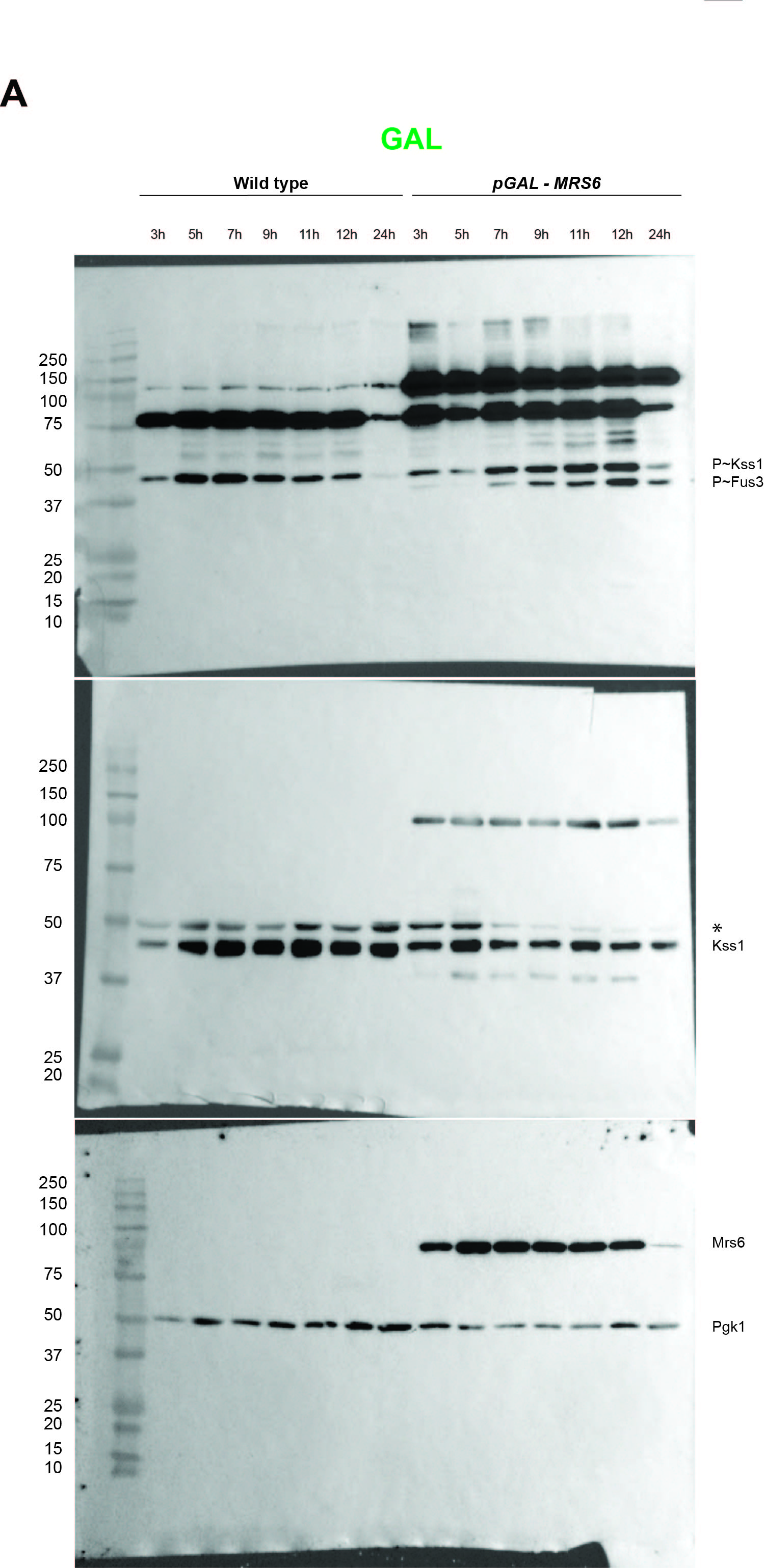

Supplement: Supplementary file 8 — Supplementary Figure s3. [file 41598_2020_78470_MOESM8_ESM.jpg]

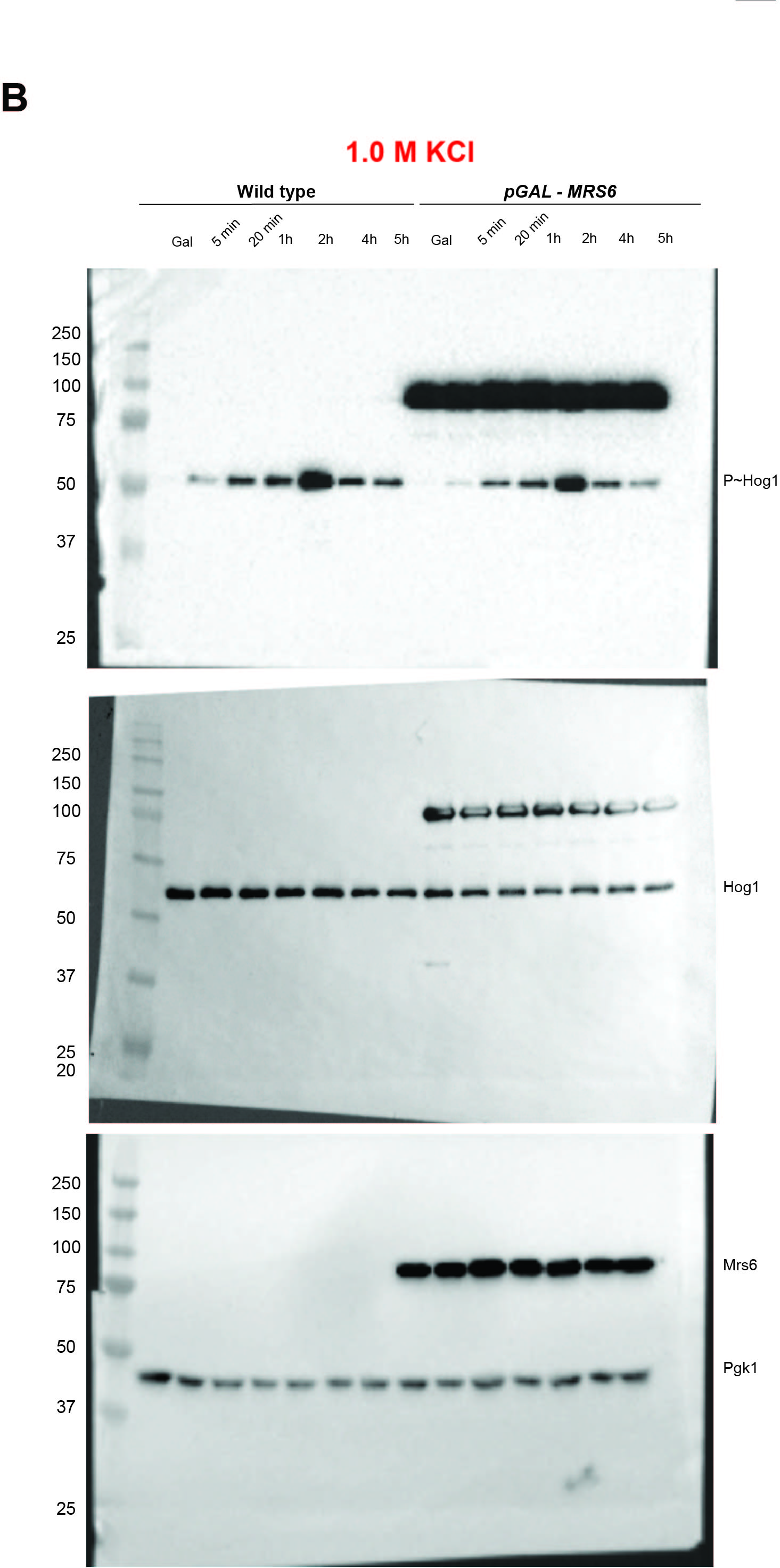

Supplement: Supplementary file 9 — Supplementary Figure s4. [file 41598_2020_78470_MOESM9_ESM.jpg]

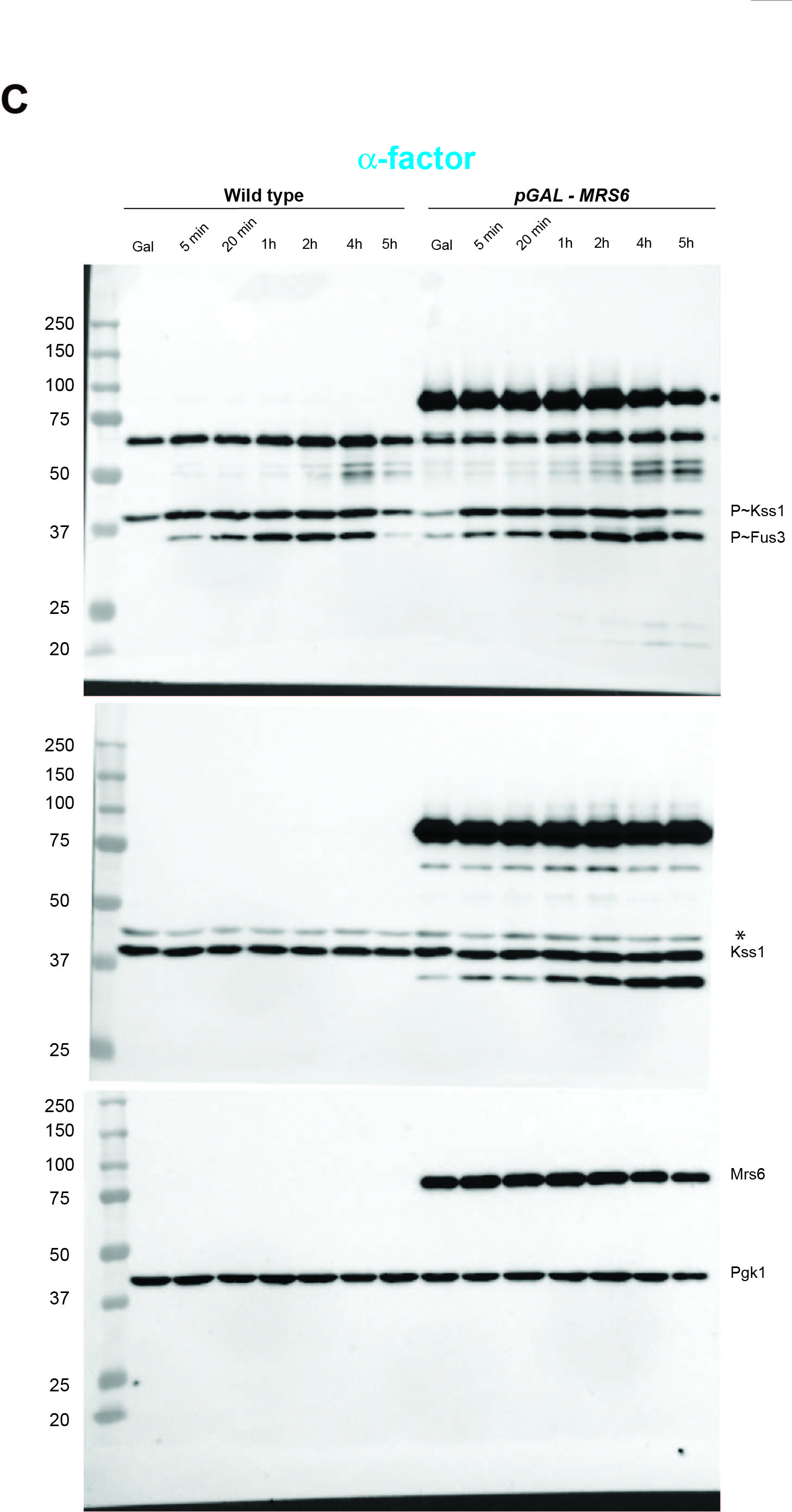

Supplement: Supplementary file 10 — Supplementary Figure s5. [file 41598_2020_78470_MOESM10_ESM.jpg]
